# Multi-Scale Analysis of the European Airspace Using Network Community Detection

**DOI:** 10.1371/journal.pone.0094414

**Published:** 2014-05-08

**Authors:** Gérald Gurtner, Stefania Vitali, Marco Cipolla, Fabrizio Lillo, Rosario Nunzio Mantegna, Salvatore Miccichè, Simone Pozzi

**Affiliations:** 1 Scuola Normale Superiore, Pisa, Italy; 2 Dipartimento di Fisica e Chimica, Università degli Studi di Palermo, Palermo, Italy; 3 Santa Fe Institute, Santa Fe, New Mexico, United States of America; 4 Center for Network Science and Department of Economics, Central European University, Budapest, Hungary; 5 Deep Blue s.r.l., Roma, Italy; Universitat Rovira i Virgili, Spain

## Abstract

We show that the European airspace can be represented as a multi-scale traffic network whose nodes are airports, sectors, or navigation points and links are defined and weighted according to the traffic of flights between the nodes. By using a unique database of the air traffic in the European airspace, we investigate the architecture of these networks with a special emphasis on their community structure. We propose that unsupervised network community detection algorithms can be used to monitor the current use of the airspace and improve it by guiding the design of new ones. Specifically, we compare the performance of several community detection algorithms, both with fixed and variable resolution, and also by using a null model which takes into account the spatial distance between nodes, and we discuss their ability to find communities that could be used to define new control units of the airspace.

## Introduction

In the recent years, network science has been largely applied to air traffic problems (for a recent review, see [1]). These studies have focused mainly on the topological characterization of the airport network [2]–[15]. In this network, airports are nodes and a link exists if two airports are connected by a direct flight. Often the number of flights between two airports in a given time window is used to weight the links, making the graph an instance of a traffic network. The interest in airport networks comes from the need of modelling traffic flow, mobility of passengers, and spreading of infectious diseases [16].

An important characteristic of a complex network is its organization in communities (clusters) [17]. Communities are generically defined as sets of nodes that are more connected among themselves than with the rest of the network. Communities are, therefore, an important element to understand and model the architecture of a network. The purpose of this paper is the identification of communities in the different networks that can be defined in the air traffic system.

Airspace is in fact a complex system which is partitioned for a series of reasons, mainly related to air traffic control. As we explain below, the European airspace is partitioned in a hierarchical way. At the highest level, the space is partitioned into multinational areas, termed Functional AirBlocks (FAB). The FABs are not yet fully implemented, but their activiation is planned in the near future as a mean to increase the capacity in terms of traffic. Then each country has its own National Airspace, which is typically partitioned into Air Control Centers. Each of these is itself partitioned into sectors, which are the smallest unit of control, being under the direct supervision of air traffic controllers. Finally, inside the sectors we find the navigation points constituting the grid where the flights move. In fact, nowadays flight plans are defined as a set of consecutive fixed points that the aircraft is supposed to pass at predefined times. On the smallest scale, therefore, a flight plan is a path on a grid whose nodes are the navigation points. The choice of the boundaries of these multiple partitions is decided in a strongly supervised and not fully quantitative way, taking into account political and strategical reasons and also traffic considerations.

To the best of our knowledge this manuscript constitutes the first attempt to apply community detection to networks of the air traffic system and also to consider different types of networks. In fact, we do not consider only airport networks but we consider three different types of network of the airspace thus creating a multi-scale structure. Beside the airport network, for which few papers [5], [18] have studied the community structure, we will consider the sector network and the navigation point network. The former is a network where nodes are sectors – the smallest units of control – and links indicate the presence of flights going from one sector to another. The latter takes the navigation points as nodes, the links being built in the same way. Making use of a unique and detailed database of the European air traffic, we investigate the topological and community properties of the sector network and of the navigation point network. To the best of our knowledge, the sector network has not been investigated before, while the topological properties of the navigation point network has been investigated only in Ref. [19] in the case of the Chinese airspace.

As detailed below, we believe that community detection in air traffic networks is important for two reasons. First, it obviously improves the characterization of the networks with respect to analysis based on the measurement of the standard metrics of network topology (degree distribution, betweenness centrality, small world effect, etc.) already considered in the literature, at least for the airport network. Secondly, and more importantly, we believe that community detection could be helpful to guide the design of new airspaces in order to have a better control of the air traffic. In particular, the Single European Sky ATM Research (SESAR) programme – which aims at a complete reorganization of the Air Traffic Management (ATM) in the next twenty years in answer to the increase of traffic in Europe – could benefit from this method.

In this paper we show how the community detection in networks provides information on the appropriateness of the airspace design at the various scales considered, based on the sole knowledge of the actual air traffic data. In this respect, the added value of this paper is twofold. On one side we show how methods devised for identifying communities in networks could be used to help designing the structure of airspace in a bottom-up way, i.e. starting from the observed behavior of the system. On the other side, this analysis could be seen as an “horse race” among different community detection methods in order to find which one works best when the investigated network describes a traffic flow (not necessarily aircraft, but also cars, data, etc.). These two aspects can not be kept fully distinct. Indeed, it is hard to distinguish whether the actual traffic flow is a consequence of the airspace partition or viceversa. As we mentioned above the airspace partition is due to different reasons sometimes unrelated with the effective air traffic needs.

The paper is organized as follows. In the next Section we present the current structure of airspace, the considered networks, and their “natural” community structure, based on the existing partitions (see also Information S1). Section “Data” describes our unique and complex database and in Section “Methodology” we present the algorithms for community detection, for comparing partitions, and to characterize the identified communities. Section “Results” presents our main results and in Section “Conclusions” we draw some conclusions.

### The Multi-Scale Network Structure of Airspace

The airspace can be considered as a multi-scale, dynamic network of interconnected entities. In this Section we give a brief overview of the airspace structure and we introduce the relevant entities for our analysis. We then describe the different networks that can be defined in the airspace and for each of them we will describe the “existing partitions” i.e. the network communities already present in the system due to the airspace partitioning in ACCs, NAs and FABs made by Eurocontrol and the air traffic service providers. Such partitions are primarily driven by the political map of Europe and by operational considerations.

### Structure of the airspace

Flights do not currently follow a smooth and optimized trajectory. Instead, they are supposed to follow a path on a predefined mesh, whose nodes are called navigation points, or *navpoints*. The position of a navpoint is given by a latitude and a longitude, but not an altitude. A flight plan is therefore a succession of navpoints, together with timestamps and altitudes, that an aircraft is supposed to follow.

The deviations from the planned trajectory are typically triggered by the air traffic controller. Specifically the airspace is divided in three dimensional airspace volumes, termed *elementary sector*, or *collapsed sector* (called simply *sector* in the following). A sector is handled by two controllers: one “separates” the aircraft (making sure they do not come too close one to each other) in the sector itself, while the other one takes care of the interface with the other sectors. The sectors are dynamic entities, which can be split or aggregated depending on the air traffic load. Moreover, the sectors can be roughly divided in two types: the en-route sectors, controlling the planes in their en-route trajectory, and the Terminal Maneuvering Areas (TMA) or the Control Zones (CTR), managing the take-off and landing phases.

The airspaces themselves are bigger, static entities aggregating several sectors. The first important one is the *Air Control Center* (ACC), where all the sectors are physically controlled from the same room (control center). In the European airspace, called ECAC (European Civil Aviation Conference), there are between 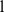 and 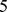 ACCs per country. Countries in the enlarged ECAC space are: Iceland (BI), Kosovo (BK), Belgium (EB), Germany-civil (ED), Estonia (EE), Finland (EF), UK (EG), Netherlands (EH), Ireland (EI), Denmark (EK), Luxembourg (EL), Norway (EN), Poland (EP), Sweden (ES), Germany-military (ET), Latvia (EV), Lithuania (EY), Albania (LA), Bulgaria (LB), Cyprus (LC), Croatia (LD), Spain (LE), France (LF), Greece (LG), Hungary (LH), Italy (LI), Slovenia (LJ), Czech Republic (LK), Malta (LM), Monaco (LN), Austria (LO), Portugal (LP), Bosnia-Herzegovina (LQ), Romania (LR), Switzerland (LS), Turkey (LT), Moldova (LU), Macedonia (LW), Gibraltar (LX), Serbia-Montenegro (LY), Slovakia (LZ), Armenia (UD), Georgia (UG), Ukraine (UK).

Then we have the *National Airspace* (NA), aggregating all the ACCs of a single country. The two dimensional boundaries of a NA are very close to the real country's boundaries. On a larger scale, we find the FABs [20], aggregating several NAs, like, for example, the Portuguese and the Spanish ones. They are not actually operative yet, but they will be important in the so-called new SESAR scenario [21], a future air traffic management scenario that will change dramatically the way air traffic is managed.

Finally, the last important element are the airports. They act as sinks and sources for the network by “absorbing” and “releasing” aircraft in the system.

### Network descriptions of the airspace

Given the structure summarized above, it is possible to define (at least) three different networks describing the airspace. The three networks operate at different spatial and temporal scales, therefore the airspace system can be considered as a multi-scale network. In order to construct the networks, we shall consider a time interval (typically one day) and we define the following graphs.

The first graph is the *network of navigation points*. In this network each node is a navigation point and two nodes are connected if at least one flight goes directly from one node to the other in the considered time interval. Similarly, the second graph is the *network of sectors*. Each sector is a node and two nodes are connected if at least one flight goes directly from one node to the other in the considered time interval. Finally the third graph is the *airport network* where nodes are airports and two nodes are connected if at least one flight goes from one node to another in the time interval.

All the networks are directed and weighted. The weight is given by the number of flights between two nodes in the given time interval. As far as the direction is concerned, we notice that most of the graphs are almost symmetric and therefore one makes a small error in considering the symmetric version of the network. Finally, note that all these networks are traffic networks. This means that the links are defined by the traffic in the time interval and are different from a street network where the infrastructure defines the link.

### Existing partitions of the airspace networks

The main objective of this paper is the comparison between unsupervised partitions of the different networks of the airspace and the partitions that are already present in the system as a result of its design. We call these partitions, *existing partitions*. Here we present the existing partitions of the three networks that we will consider in the following. A set of Figures with the different existing partitions for the European airspace is shown in Information S1.

The navpoint network can be partitioned in terms of national airspaces (see Fig. S1) or in terms of control centers (see Fig. S2). The sector network can be partitioned in terms of FABs (see Fig. S3 and Table 1 in Information S1), in terms of national airspaces (see Fig. S4), or in terms of control centers (see Fig. S5). It is worth mentioning that the considered 12 FABs include the 9 FABs planned by ECTL plus other three FABs defined by the authors and based on geographic proximity with the purpose of appropriately comparing the existing and obtained partitions. Finally the airport network can be partitioned in terms of FABs (see Fig. S6) or in terms of national airspaces (see Fig. S7).

**Table 1:** Average number of communities in the network of navpoints and average minimum number of communities containing 90% of the nodes.

Existing part.	ACC	NA			
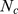	75.5 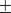 2.6	46.0 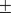 0.			
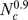	53.3 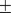 2.2	20.0 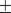 0.			
Unsupervised part.	Inf.	Modularity	OSLOM	Mod. max.	Mod. max.
				( 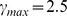 )	( 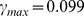 )
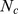	598.0 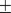 14.7	42.8 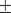 3.9	150.0 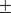 21.6	63.1 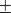 4.6	44.2 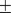 3.5
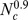	408.8 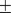 8.5	25.9 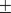 1.6	75.1 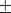 4.4	36.3 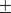 2.2	24.6 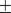 1.6

The top part refers to existing partitions and the bottom part to the unsupervised partitions. Results were obtained averaging over 28 days, with standard deviations taken as error bars.

### Data

Our database contains information on all the flights that, even partly, cross the ECAC airspace. Data are owned by EUROCONTROL (http://www.eurocontrol.int), the European public institution that coordinates and plans air traffic control for all of Europe. Data were obtained as part of the *SESAR Joint Undertaking* WP-E research project ELSA “Empirically grounded agent based model for the future ATM scenario” after competing in a 2011 public call issued by SESAR Joint Undertaking. Data can be accessed by asking permission to the legitimate owner (EUROCONTROL). The owners reserve the right to grant/deny access to data.

The data come from two different sources. First, we have access to the Demand Data Repository (DDR) [22] from which we have all the trajectories followed by any aircraft in the ECAC airspace. In this paper we consider a 28 day time period (termed AIRAC cycle), specifically the one lasting from the 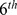 of May 2010 to the 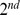 of June 2010. This AIRAC cycle is not including major holidays that might alter the estimation of statistical properties. A trajectory, called indifferently flight plan here, is made by a sequence of navigation points crossed by the aircraft, together with altitudes and timestamps. The typical time between two navpoints lies between 1 and 10 minutes, giving a good time resolution for trajectories. In this paper we only use the “last filed flight plans”, which are not the real trajectories flown, but the planned trajectories – filed from 6 months to one or two hours before the real departure. We do not use the real trajectories because we do not want to include other factors of disturbances, like weather, in our analysis. We selected only scheduled flights – excluding, in particular, military flights – using landplanes (regular aircraft) and having a IATA code. This gives, in first approximation, the set of commercial flights. We also excluded all flights having a duration shorter than 10 minutes and a few other flights having obviously data errors.

The other source of information are the NEVAC files [23] that contain all the elements allowing the definition (borders, altitude, relationships, time of opening and closing) of the elements of airspaces, namely airblocks, sectors, airspaces (including FIR, National Airspace, ACC, etc.). The active elements at a given time constitute the configuration of the airspace at that time. These files allow to determine the configuration of the airspaces for an entire AIRAC cycle. Here we only use the information on sectors, airspaces and configurations to rebuild the European airspace. Specifically, at each time we have the full three dimensional boundaries of each individual sector and airspace in Europe.

## Methods and Materials

### Community detection methods

In this article, we consider different algorithms of community detection on networks. Specifically, we consider Infomap [24], the maximization of the modularity with the Louvain method [25] and simulated annealing, and OSLOM [26]. Moreover, since we want to identify the hierarchical structure of airspace communities, we also used the multi-resolution modularity [27]. This method is also useful to investigate the robustness properties of the modularity partition (see below and [28]).

The first algorithm we used is called Infomap [24]. The idea behind the method is to consider a random walk over the network. The more the nodes are connected one with each other, the more the walker will stay with them and thus form a community. The analysis of the flows over the network gives access to the underlying community structure. More precisely, the algorithms compute an optimized compressed description of information flows and, from the information theory point of view, the community detection algorithm searches the partition which minimizes the description length of an infinite random walk over the network. Numerical experiments [17] indicate that this algorithm has a complexity 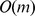 where 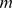 is the number of edges. It is thus efficient with sparse networks, where 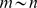 with 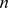 the number of nodes. In our investigation we used the usual implementation of the package, available online [29].

The second method is based on the maximization of the modularity. For a given partition 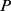, the modularity 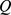 is the sum of the number of links within each community minus the expected number of links for a given null model, i.e.
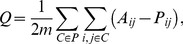
(1)where 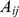 is the element 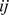 of the weighted adjacency matrix of the graph, 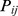 is the element 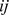 of the weighted adjacency matrix under the null model, and 
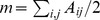
 is the total weight in the network. The most popular choice for 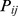 is the one proposed by Newman and Girvan (NG) [30]: 
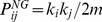
, where 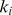 is the strength of node 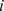. The null hypothesis corresponds to a randomization of the links preserving the strength of each node. It is well known that modularity has a resolution limit [31], i.e. in large networks modularity fails to resolve small communities.

For this reason, we also considered the multi-resolution modularity [27], which gives the possibility of spanning different resolutions. In fact, by replacing 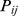 by 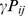 in Eq. 1 one gets
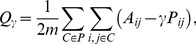
(2)and one can easily span different levels of description by changing the resolution parameter 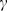. By increasing 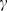 one typically obtains smaller and smaller communities, until one gets each node in its own separate community. On the contrary, decreasing 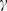 eventually leads to a single community including all the nodes of the network. Multi-resolution modularity gives also information on the robustness of the identified communities. In fact, when the identified communities are essentially unchanged when the resolution parameter is varied inside an interval, one can conclude that the partition is robust. This is signaled by the presence of plateaus in the curve describing the number of communities as a function of 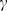.

Modularity can also be generalized by using different null models. In Section “The airport network”, we examine a null model which takes into account the spatial localization of the nodes. Following Ref. [32], we use for 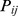 the following form:
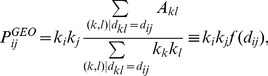
(3)which is the weighted probability for a node 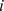 to be linked to another node 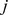 at euclidean distance 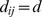. This choice allows to see the communities which are not only explained by their geographical proximity. It is important to notice that, differently from the gravity-like models where the distance function is assigned a priori, here we use the actual data to infer the deterrence function 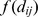 (as done also in Ref. [32]) describing the role of distance between nodes in determining the probability that they are linked according to the above Eq. 3.

Different computational methods can be used to find the maximum of modularity. One of the most popular is the Louvain method, an algorithm which computes the communities, then the induced graph – where each node is a community – then the communities of this graph, until a maximum in modularity is reached. This method is very efficient since the complexity is numerically estimated to be 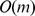 [25] and it gives accurate results. We used the software package available at [33]. However, this cannot be used straightforwardly when the null model is the one of Eq. (3). Since the probability needs explicitly some geographical coordinates, computing the induced graph is meaningless, because one cannot associate each node (community) with spatial coordinates.

Instead, when using the null model of equation Eq. 3, we choose a simulated annealing method. The simulated annealing, based on a physical process used to change the properties of glass or metals in the industry, is typially used to find a minimum (or maximum) of a non monotonic multi-dimensional function. It is based on a random walk in the phase space: at each step, one changes slightly the system (here for instance, changing the assignment of a node to a community) and see if the function to be optimized (the “energy”) has decreased or increased. Given that we look for a minimum here, we always accept the new state in the first case (decrease), and randomly choose if we accept the new state in the latter case (increase). The most widely chosen probability of acceptance is usually of the form 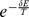, where 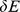 is the change in the function between the new and the previous state, and 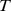 is a parameter called temperature, in analogy with the physical process. The simulated annealing algorithm itself consists in a progressive decrease of 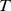, so that the system first explores big wells of energy, then progressively gets trapped in deeper, narrower wells. The algorithm gives accurate results but needs much more time to converge than the previous methods. As a final comment, when applying the modularity partition (with the Louvain method or with the simulated annealing) we considered the undirected version of the networks. As mentioned above, air traffic networks are highly symmetric and therefore the error should be small.

The last method of community detection we use is called OSLOM – for Order Statistics Local Optimization Method [26]. Its general principle is the following. Using the NG null model presented in the standard definition of modularity, the authors use a fitness function – based on the probability that an external node to a community has a given number of neighbors within this community – to assess the statistical significance of each community. More precisely, each external node is ranked following this fitness function, and the algorithm tests the likelihood of the score, with the given rank of this node against the null model. This procedure has several benefits. Since this optimization is local, i.e. made independently for each community, the result can be a partition with overlapping clusters. In our analysis we found very few nodes belonging to two or more communities, and they have typically small degree, strength, or centrality. To simplify our analysis we assigned them randomly to one of the communities. Moreover, the method can be used as refinement to other methods (Infomap, modularity), because one can give to the algorithm an existing partition as input. The method's complexity itself cannot be exactly computed, but numerical results shows that it is close to 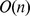. The package is available at [34].

### Assessing the robustness of communities

When performing community detection in networks it is important to assess the robustness of the obtained partition. In fact, community detection algorithms give always an output, even when the investigated network has no community structure. The output is not necessarily a partition but it might be a unique community. In both cases, one needs to perturb the system and test whether the partition is robust with respect to this perturbation.

There are different ways of assessing the robustness of a partition. As suggested for example in Ref. [28], the robustness of a partition is usually assessed by sweeping a resolution parameter, changing the (random) optimization, or by slightly perturbing the graph structure and measuring the change in the outcomes, to assess the robustness of the outcomes of each individual algorithm (see also [35], [36]).

As mentioned above, in this paper we include the multi-resolution modularity in our detection methods. The presence of plateaus in the number of detected communities as a function of the resolution parameter 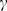 can therefore be considered as a robustness analysis (see also [28]).

Moreover we also use a direct test of robustness obtained by perturbing the graph structure. Following [36], given a network and an initial partition (given by a method), we create 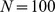 replicas of the network by randomly adding 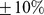 to the weight of each link. We then compute the adjusted, normalized mutual information (see next Section) between each of the replicas and the initial partition. Then we define robustness as the average of this value over all replicas. Thus, a score close to 1 for robustness indicates that the communities found by the given method are a real structure of the network. On the contrary, if the score is low, then the communities found can be viewed as computational artifacts.

Finally, it is important to notice that for our specific system we have one network per day. Since the community structure of the airspace should not change significantly from one day to the next, comparing partitions of different days can be considered another method of performing robustness analysis. In fact, given a type of network and an algorithm, if the detected communities are similar across different days, we can consider this result in support of the robustness of the identified communities.

In conclusion, we perform three robustness tests: multi-resolution modularity (by detecting the plateaus), graph perturbation (by computing the Mutual Information over replicas), and comparison of partition across days (by computing the standard deviations over days with existing partitions).

### Metrics for comparison of partitions

In order to compare the partitions given by the different algorithms, we used two different metrics. The first one is called the Rand index (RI) [37] and is computed in the following way. Given two different partitions 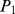 and 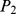 of the same set and a pair of elements in this set, there can be four cases: a) the elements are in the same community in 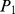 and 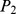, b) they are in different communities in 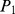 and 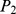, c) they are in the same community in 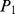 but in different ones in 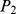, and d) they are in the same in 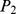 but not in 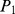. The Rand index is simply the number of occurrences of the two first cases a and b over the total number of possibilities. The Rand index ranges between 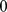 and 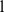.

The second metric is the mutual information (MI). The mutual information between two random variables 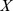 and 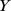 is
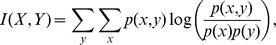
where 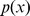 and 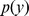 are the marginal probabilities to have 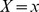 and 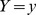 and 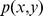 is the joint probability. The mutual information is symmetric. This definition can be used to compare partitions, where 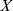 and 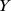 represent the labels of communities in each partition.

Since these two metrics can have non-zero values for two purely random partitions, due to the finite size of the sample, we use an adjusted value for each metric. Moreover, in order to be able to compare the values for different variables, we normalize both metrics. Thus, we divided each adjusted metric by their maximum value. Specifically, given two partitions 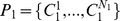 and 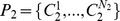 of a set of 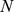 elements, 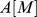 denotes the adjusted, normalized version of 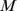, with 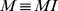 (mutual information) or 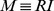 (Rand index).

Given a null model describing the random partitions, 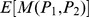 is the expected value of 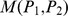. The adjusted, normalized version of 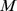 is
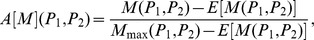
where 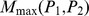 is the maximum value of 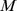, obtained when the two partitions are identical.

Defining 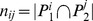 the number of common elements in communities 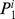 and 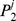, 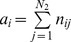, and 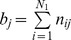, these maximum values are
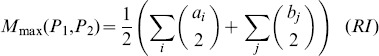


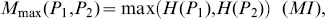
with 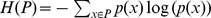 the entropy of the partition 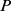.

As a null model for computing the expectation 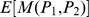 we use an hypergeometric model. Therefore the expected value of the Rand Index is
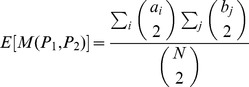
and the expected value of the Mutual Information is
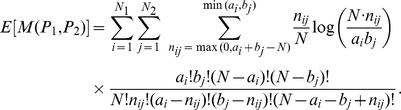



After being adjusted and normalized, RI lies between −1 and 1 and MI lies between 0 and 1. The value 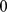 implies that the two partitions have no more in common than two random partitions would have. The value 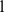 implies that the two partitions are exactly the same.

In the following we will use RI and MI to compare the existing and unsupervised partitions in each day. In the Tables we report the average and standard deviation of these metrics computed across the 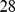 days of the investigated AIRAC cycle.

### Community characterization

After a network has been partitioned into communities, the next step is to characterize the nodes composing each community. Characterization of a community means to identify which characteristics of the nodes are “over-expressed” [38]. When we will consider the partition of the network of airports (see Section “The airport network”) we will give to each node (airport) an attribute indicating the country the airport belongs to. Then we will ask the question of which countries, if any, are over-expressed in each community.

Let us consider a network where the nodes have all an attribute 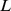 which can take several values 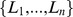. For each attribute 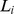, we want to see if it is over-expressed in a given community 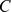. The probability of having 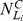 nodes with attribute 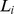 in a community 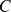 composed by 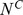 nodes under the null hypothesis that elements in the community are randomly selected is given by the hypergeometric distribution [38], i.e.
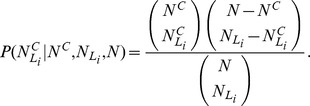
where 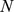 is the total number of nodes. Then the probability that 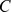 has more than 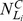 nodes with attribute 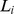 is given by 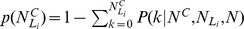. If this probability is below a given threshold 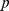, then 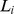 is said to be over-expressed in 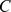, because it is unlikely to find in 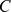 such a large number of nodes with this attribute only by chance. Since we are performing several tests on the different values of 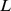 and on different communities, the value of 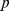 has to be corrected in order to avoid a large number false positives, because of the multiple tests performed. One way of dealing with multiple hypothesis tests is to set the threshold to 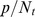, where 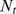 is the number of tests. This multiple hypothesis test correction, known as the Bonferroni correction, is the most conservative, but other corrections can be found in the literature [39].

## Results

### The navigation point network

The navigation point network is the most detailed network we consider in this paper. To the best of our knowledge, this type of network has been investigated only in Ref. [19], where some basic network metrics for the Chinese airspace have been studied. Before discussing the community detection result, we present some network metrics for the European case.

#### Network metrics

The navpoints are defined with a longitude and a latitude, but without an altitude. As a consequence they can be used independently by several sectors at different altitude. Thus, a navpoint cannot be considered as a single entity/node in terms of the network. On the other hand, the structure of the airspace has an interesting property which is of use here. Indeed, three phases are usually defined for trajectories of flights: the take-off, the en-route, and the landing phase. If one considers only the en-route part, the corresponding portion of airspace becomes much simpler. At high altitudes, the navpoints are shared only by one or two sectors stacked vertically, and these sectors have often the same 2D boundaries. Thus, we chose to consider only the upper part of all the trajectories, cutting them below 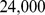 feet – the value considered to indicate the end of the takeoff phase or the beginning of the landing phase. Moreover during takeoff and landing flights are often allowed to use ‘temporary navigation points” i.e. to deviate also significantly from the navpoint network, in order to allow a better control of the high traffic around airports. This way, navpoints are contained in only one or two sectors and are meaningful entities.

**Figure 1:**
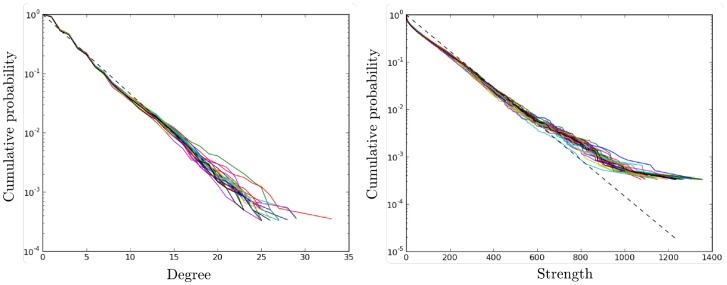
Distribution of degree (left) and strength (right) in the navigation point network in a semilog scale. Each color corresponds to a different day of the AIRAC cycle. The mean degree is 3.88 with standard deviation 3.0, with min and max respectively equal to 1 and 29. The mean strength is 87.0 with standard deviation 118.7 and min/max equal to 1 and 1338. The dashed line indicates the best exponential fit of the average curve.

Based on these trajectories, we build the navpoint network. Each of them is a node and a link exists if at least one flight goes from one to the other in the given time-frame (one day). The link is weighted with the number of flights. For the whole ECAC space, the network has around 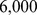 nodes, and the 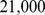 flights per day create 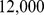 edges. The distribution of degree and strength of the navpoint network are presented in Fig. 1. The distribution is quite stable in different days and it is very close to an exponential. This is an expected behavior, since this network is strongly constrained geographically. Indeed, an aircraft cannot skip many navigation points to go from one point to another, and is bound to travel through geographical neighboring points.

#### Communities

We now consider the communities formed by the traffic on the navpoint network. Specifically, our aim is to see if the bottom-up clusterizations made by the algorithms are consistent with the top-down design which led to their creation. Even though the details of the partitions depend obviously on the traffic demand, they are not at the root of the design. Instead, by using unsupervised clustering, we do not have any prior on the partition, other than the boundaries of the ECAC space. In this sense, this approach is complementary to the existing top-down “expert” partitioning and can give insights on how to improve it. We use the different methods presented in Section “Methodology” to generate the partitions and compare them with the existing partitions based on the NA and the ACC (see Information S1) by using the metrics presented in Section “Metrics for comparison of partitions”.

**Figure 2:**
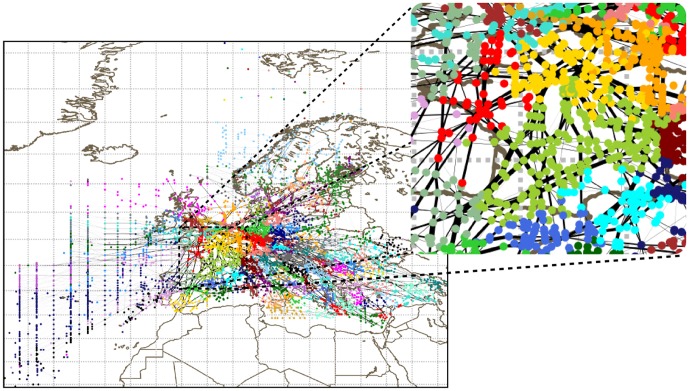
Communities obtained with the OSLOM algorithm on the network of navpoints for May 6, 2010. Each color corresponds to a different community.

Fig. 2 shows one of these partitions, obtained with the OSLOM algorithm. The clusters are clearly geographical, with almost no geometrical overlap between them. For this algorithm, communities are much smaller than the national scale, but their boundaries seem roughly consistent with the national boundaries.

We perform an extensive community detection by using the three algorithms described in Section “Methodology” for each of the 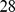 days of the AIRAC cycle. In the case of the modularity-based algorithms we consider 
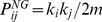
. The average number of communities for each algorithm is reported in Table 1. We notice that the number of communities depends significantly on the adopted algorithm. The coarsest level of description is given by the modularity maximization, with only 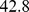 clusters, even less than the number of countries. OSLOM gives more than three times this number (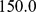), and twice the number of ACCs (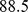). Finally, Infomap gives around 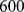 communities, a very large number, comparable only to the number of sectors. The tendency of Infomap to give small communities when their structure is non-clique like, like in geographically embedded networks, has been explained in Ref. [40]. In the second line of Table 1 we show the minimum number of communities (induced or existing) which involve 90% of the nodes in the network. These Figures indicate that a fraction of the communities ranging from 30% (ACC) to 50% (OSLOM) contains only 10% of the nodes. These are therefore very small communities. It is interesting to note that such small communities are present also in the existing partitions, indicating that there is a significant heterogenity in the size of the existing communities.

**Figure 3:**
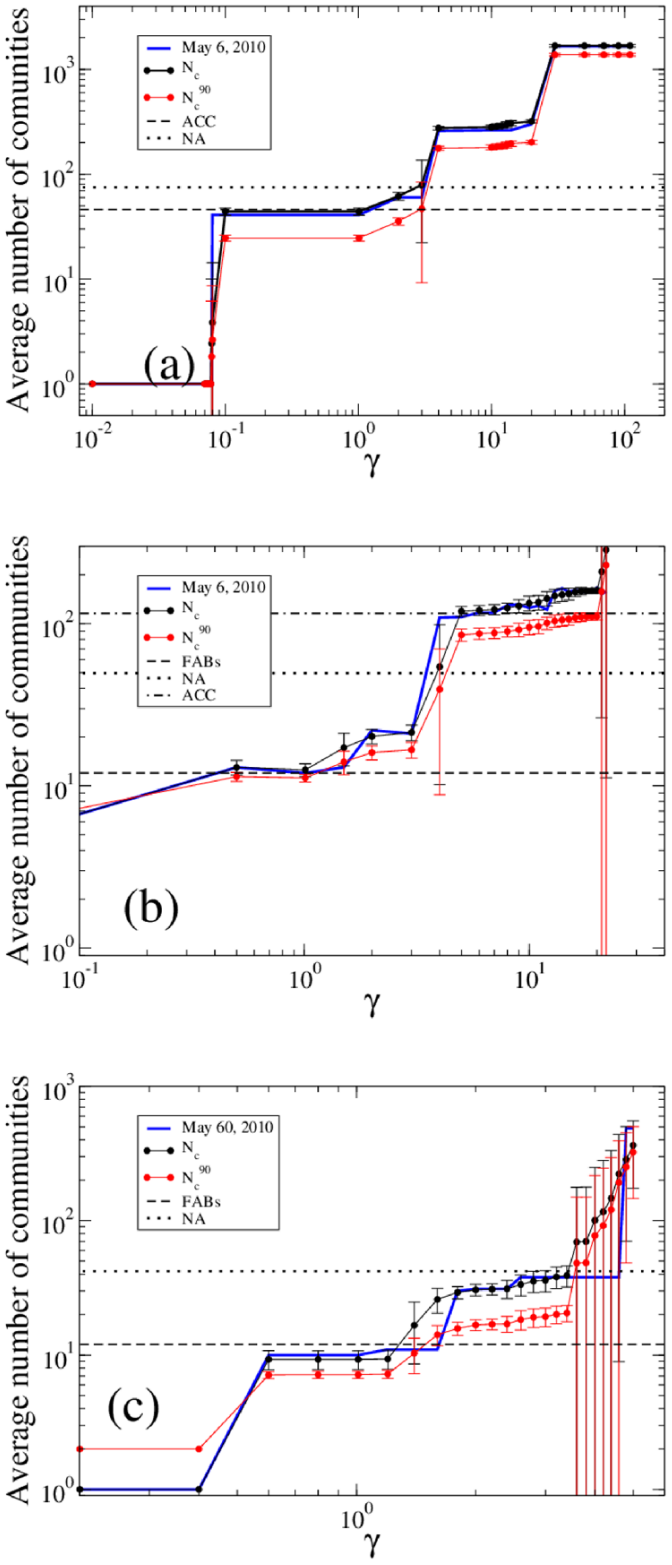
Number of communities in the three networks obtained with multi-resolution modularity as a function of the resolution parameter 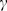. Panel (a) refers to the navpoint network, panel (b) to the sector network, and panel (c) to the airport network. The blue line refers to one specific day, namely May 6, 2010. The black circles are the average number of communities and the red circles are the average minimum number of communities containing 90% of the nodes. Averages are taken over the 28 investigated days. Error bars are standard deviations. The horizontal black lines are the average number of communities in the existing partitions. Note that plots in panels (a) and (b) are not displaying the last plateau, corresponding to the case where each node is in its own community.

It is clear that different algorithms are able to identify communities at different spatial scales. The multi-scale structure of the navpoint network is well captured by the multi-resolution modularity. Panel (a) of Fig. 3 shows, as a function of 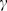, the number of communities identified by the maximization of the modularity 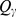 of Eq. 2 on May 6, 2010. We also show the number of communities averaged on the 28 days of the AIRAC cycle, for each value of 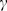. Added to this, for each obtained partition we computed the minimum number of communities which include 90% of the nodes. We also show the average of this number on the plot. It is important to notice that the maximization of 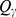 produces very clear plateaus corresponding to a number of communities equal to 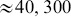, and 2,000. While the first is easily comparable with the number of NAs, the other two could represent control units of the airspace on a smaller scale. Moreover there is probably a small plateau corresponding to 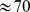 communities, and this number is very close to the number of ACCs (see also below). Finally notice that the error bars, taken as standard deviations of the number of communities in the AIRAC cycle, are very small, indicating that the communities are very robust.

In order to compare the multi-resolution modularity partitions with the other three partitions, we select the values of 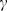 that give partitions maximizing the MI with ACC and NA partition. These correspond to 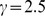 and 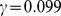, respectively. Note that these values correspond to the average *beginning* of the plateaus. Hence, 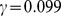 for instance corresponds to the same plateau than 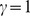. So these values of 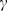 should be considered as labels and have no intrinsic importance. From the last two columns of the bottom part of Table 1, we observe that the number of communities of these partitions are close to the real ones, even if systematically underestimated.

To make the comparison between existing and unsupervised partitions more quantitative, we use the two metrics described in Section “Metrics for comparison of partitions”. The partitions have been computed over the 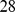 days of the AIRAC cycle and the MI and RI averaged on this period. The results of this systematic comparison between partitions are given in Table 2. We find that the Infomap partition is the most different with respect to the existing partitions. This result is somehow expected, because the Infomap communities are many more and smaller than those obtained with the other algorithms. The other two induced partitions are roughly equally close to the existing partitions. More precisely, OSLOM is closer to the ACCs partition and modularity to the NA partition, as expected from the number of communities. By using the multi-resolution modularity we are able to achieve partitions which are even closer to the existing partitions. As Table 2, the partition obtained by maximizing 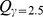 is the closest to ACC partition, both considering MI and RI. Similarly the partition obtained by maximizing 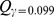 is the closest to NA partition, both considering MI and RI.

**Table 2:** Comparisons of the partitions of the navpoint network by using the Rand index, the Mutual information, and the robustness.

Rand Index	Infomap	Modularity	OSLOM	Mod. max. ( 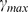 )	
ACC	0.078 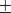 0.003	0.28 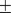 0.02	0.28 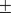 0.02	**0.29** 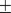 **0.03**	2.5 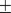 0.5
NA	0.043 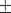 0.002	**0.30** 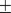 **0.03**	0.23 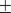 0.02	**0.30** 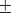 **0.03**	0.099 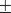 0.005
**Mutual Inf.**	Infomap	Modularity	OSLOM	Mod. max. ( 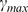 )	
ACC	0.37 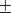 0.004	0.57 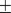 0.01	0.59 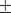 0.01	**0.61** 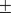 **0.01**	2.5 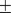 0.5
NA	0.32 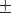 0.003	**0.57** 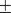 **0.01**	0.51 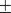 0.09	**0.57** 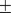 **0.01**	0.099 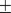 0.005
**Robustness**	Infomap	Modularity	OSLOM	Mod. max. ( 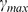 )	
ACC	**0.90** 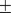 **0.01**	0.79 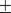 0.01	0.82 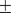 0.01	0.75 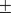 0.04	2.5 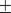 0.5
NA				0.78 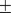 0.01	0.099 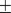 0.005

The numbers are the average values, over the 28 days of the AIRAC cycle, between the partitions and the error bars are standard deviations. Numbers in boldface refer to the partition that maximizes the corresponding metric.

The issue of the robustness of the obtained partitions is already addressed for the multiresolution modularity, because we find clear plateaus. However, we have performed the randomization experiments described in Section “Assessing the robustness of communities” for the other partitions (Infomap, etc.). The results are shown in the bottom part of Table 2. We observe that all the methods give quite high values of robustness, strongly suggesting that the detected partitions are robust to perturbations of the amount of traffic in the network. This result is also supported by the relatively small standard deviation of the number of communities, MI, and RI obtained by considering the different 28 days of our sample. The Table also shows that Infomap gives the most robust partitions, and this is maybe due to the small size of its communities. The partitions with the other methods have similar robustness properties. Finally, we notice that the standard errors shown in Tables 1 and 2, for the number of communities but also for their metrics comparing the partitions, are small compared to the means. This means that the partitions are quite robust across days, thus providing another indication of the robustness of the unsupervised partitions.

All these results underline the difference from the bottom-up approach with an unsupervised partition based on the traffic and the top-down approach of the real construction of the existing partitions. The next Section deals with the same idea at a coarser level: given some predefined partitions of the sectors, we ask how these clusters can be aggregated based on the traffic from one sector to the other.

### The sector network

The sectors are the smallest operational pieces of airspace and as such are controlled by a pair of controllers. One in particular is in charge of the interaction with the other neighboring sectors. Thus, building the network of sectors, where nodes are sectors and links are built from traffic data between two sectors, gives the finest description of the operative European airspace. To the best of our knowledge, this network has never been studied in the literature. As for the navpoint network, we present first some basic network metrics and then we move to the community detection problem. Here, we used the full network of sectors, without cutting the trajectories in altitude, and without discarding the airports areas. Moreover since sector are dynamic entities that can be split and merged during that day, responding to traffic variation, and because we want to use all the traffic data and not just a snapshot at a given time, we decided to include in the network all the sectors active during each day. For instance, if a sector is sometimes split in two parts, our network will contain three nodes: one for the sector and one for each part.

#### Classic metrics

The number of active sectors in Europe varies from 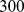 (around midnight) to more than 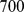 during the day. In order to build the network, we considered that there is a link between two nodes if a flight goes directly from one sector to the other in the considered time window. The links are also weighted as before, i.e. by using the number of flights.

**Figure 4:**
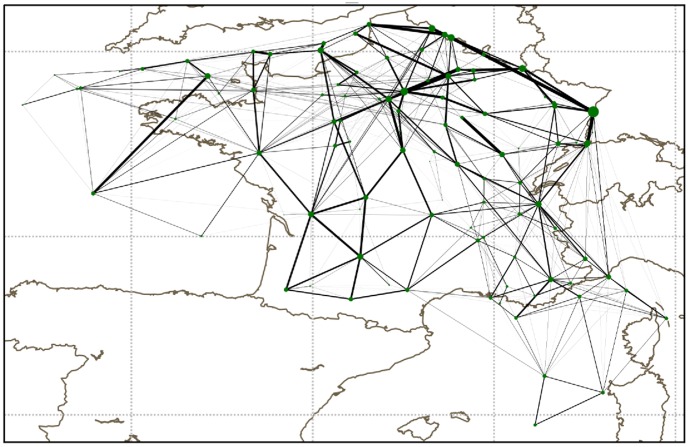
Example of the sector network for the French airspace. Each node represents a sector, the thickness of the links between them is proportional to the number of flights passing directly from one sector to the other. Sectors which are exactly on top of each other are a bit shifted to see them.

**Figure 5:**
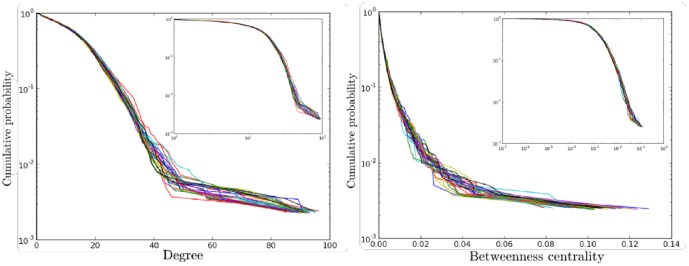
Distribution of degree (left) and node betweenness centrality (right) of the sector network of the European airspace in semi-log scale (insets: in log-log scale). Each color corresponds to a different day of the AIRAC cycles. The mean degree of the network averaged on 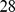 days is 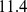, with a standard deviation equal to 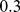. The intraday standard deviation is however very large, being equal to 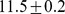. The network is thus heterogeneous, even if not scale free. The corresponding values for strength are 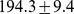 for the mean and 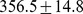 for the standard deviation in one day. Finally the betweenness centrality displays a averaged mean degree of 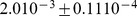 and a standard deviation of 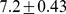.

An example of the sector network of the French airspace is given in Fig. 4. For the whole Europe, the network has around 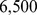 links per day. The degree distribution is displayed in the left panel of Fig. 5. As one can see, the distribution displayed is distinct from a power law, but also from an exponential. We notice here the presence of very few hubs (mostly German sectors), i.e. central sectors which redistribute the traffic around Germany in many other sectors. The strength distribution (not displayed here, but available upon request) is a bit closer to an exponential. The betweenness centrality, shown on the right panel of Fig. 5, does not seem to be a power law nor an exponential. In fact, the network is far from being homogeneous and the standard deviations computed within a day over the nodes are of the same order or greater than the average degree, strength, or betweenness, which means that the distributions are very wide.

These characteristics and the fact that the distribution is different from a power-law distribution, are the results of geographical constraints. Indeed, even if the network is not exactly planar, there is no “short-cut” between far away sectors. An aircraft has to go through a sequence of nodes to reach the destination. This implies a node is more likely to be connected to its geographical neighbors, which are of course of limited number.

#### Communities

We use the same methods described earlier for the detection of communities in the network of sectors and we compare them with three existing partitions, namely the ACCs, the NAs, and the FABs (see maps of Figs. S3, S4, and S5 in the Supporting Information).

**Figure 6:**
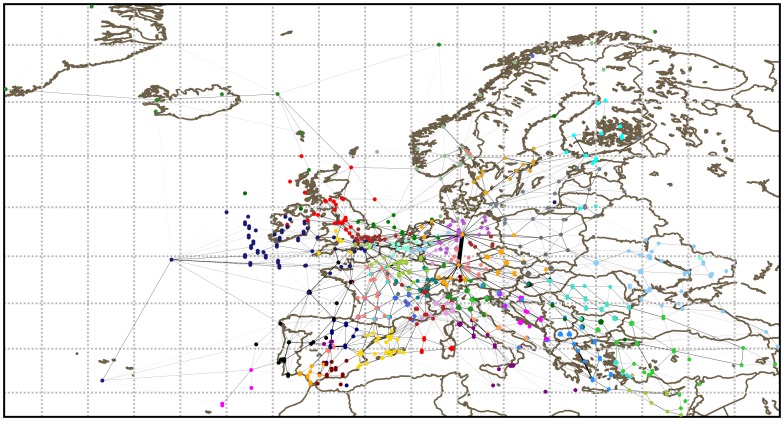
Community detection in the European network of sectors using the Infomap algorithm. The network is computed on the May 6, 2010. Each color corresponds to a different community.

Fig. 6 shows the result of the Infomap method on this network. The detected communities are typically much smaller than the typical size of a country. However it is interesting to notice that they are not transnational either, and thus they seem to partition the national airspaces themselves. Moreover, some known specificities of the European airspace are well recovered. For example, the two big communities of Ireland and North United Kingdom are present, as well as the four ACCs of Italy (see Fig. S5 in Supporting Information).

Table 3 gives the number of communities for each partition, averaged across days. Since the number of communities is related to their typical size, we notice that the average size of the communities is quite different from one algorithm to the other. The modularity method, due probably to resolution effects (see Section “Methodology”), gives the biggest clusters and their numbers are close to the number of FABs. On the contrary, the OSLOM and Infomap give smaller clusters, of the typical size of a country. These considerations, based on the sole number of communities, are clearly not sufficient to compare different partitions, because, as we can see from Fig. 6, the community sizes are quite heterogeneous over Europe. In the second row of Table 3 we show the minimum number of communities (unsupervised or existing) that involve 90% of the nodes in the network. As for the navpoint network, also here there are very small communities. However, the number of small communities is here smaller than in the previous case. This might be an indication of the fact that sectors are more inter-connected than navpoints. The only obvious exception is given by the NA case, where now almost 50% of communities contains 10% of nodes while such percentage was 30% in the case of the navpoint network.

**Table 3:** Average number of communities in the network of sectors and average minimum number of communities containing 90% of the nodes.

Existing part.	ACC	NA	FABs			
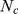	115.4 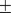 2.0	49.5 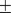 0.7	12.0 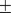 0.			
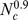	58.7 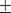 0.8	24.0 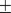 0.	9.0 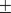 0.			
**Supervised part.**	**Inf.**	**Mod.**	**OSLOM**	**Mod. max.**	**Mod. max.**	**Mod. max.**
				( 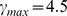 )	( 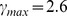 )	( 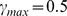 )
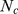	66.7 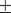 3.3	12.6 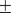 1.1	49.9 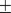 3.0	86.9 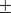 45.6	24.0 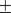 17.8	13.0 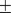 1.3
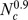	42.9 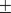 3.0	11.1 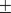 0.7	39.5 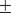 2.5	62.4 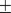 31.9	18.5 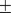 12.2	11.4 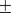 0.7

The top part refers to existing partitions and the bottom part to the unsupervised partitions. Results were obtained averaging over 28 days, and the errors bars are standard deviations. The number of ACCs is not stable because some of them are not used during the period.

As for the navpoint network, we find the values of 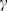 in the multi-resolution modularity such that the obtained partition has the maximum MI with the three existing partitions. We obtain 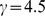, 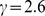, and 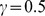 as the values that maximize the MI with ACC, NA, and FABs, respectively. The number of communities of these partitions are quite close to the real one, especially when considering communities accounting for more than 90% of the nodes. Also in this case, the analysis of the number of communities obtained by maximizing 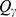 as a function of the resolution parameter 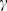 reveals the multi-scale structure of the sector network. Panel (b) of Fig. 3 shows that the number of communities has clear plateaus corresponding to a number of communities equal to 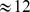 and 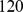, very close to the number of FABs and ACCs. Like for the navpoint network, the panel does not show the last plateau, corresponding to each node in its on community. On the other hand no plateau is observed for a number of communities close to the number of NA, suggesting that the division in national airspaces has no motivation in terms of traffic, but only political ones. Indeed, a small plateau is observable between the main ones, but very close to the first one (FAB).

A more detailed comparison between all these partitions is presented in Table 4. As before, for multi-resolution modularity we select the three values of 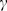 that maximizes the mutual information with the three existing partitions. A more mixed pattern emerges for sectors. For ACC, the closest partition is the one generated by OSLOM (according to RI) or Infomap (according to MI). Modularity or multi-resolution modularity give instead the closest partition to NA and FABs according to both metrics. In the case of FABs the modularity and multi-resolution (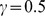) partitions have a similar average number of communities with respect to FABs. However the MI value is relatively small in both cases. Therefore, it is worth emphasizing that their boundaries are very different from the FABs, and this suggests a possible alternative design to the FABs which preserves approximately the total number of communities.

**Table 4:** Comparisons of the partitions of the sector network by using the Rand index, the Mutual information, and the robustness.

Rand Index	Infomap	Modularity	OSLOM	Mod. max. ( 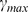 )	
ACC	0.38 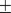 0.03	0.21 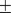 0.02	**0.42** 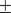 **0.03**	0.25 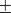 0.03	4.5 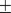 1.2
NA	0.27 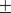 0.02	**0.37** 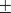 **0.03**	0.32 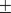 0.03	0.36 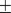 0.04	2.6 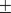 0.8
FAB	0.17 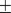 0.01	**0.32** 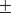 **0.03**	0.20 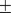 0.02	**0.32** 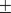 **0.03**	0.5 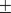 0.1
**Mutual Inf.**	Infomap	Modularity	OSLOM	Mod. max. ( 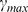 )	
ACC	**0.58** 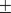 **0.01**	0.39 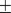 0.01	**0.58** 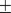 **0.01**	0.47 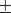 0.02	4.5 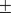 1.2
NA	0.52 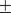 0.01	0.53 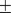 0.02	0.55 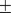 0.02	**0.58** 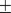 **0.02**	2.6 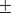 0.8
FAB	0.40 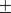 0.01	**0.54** 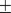 **0.02**	0.43 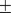 0.01	**0.54** 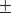 **0.02**	0.5 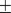 0.1
**Robustness**	Infomap	Modularity	OSLOM	Mod. max. ( 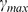 )	
ACC	**0.90** 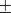 **0.02**	0.78 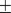 0.02	0.67 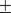 0.06	0.74 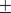 0.04	4.5 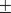 1.2
NA				0.74 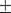 0.03	2.6 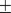 0.8
FAB				0.77 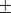 0.03	0.5 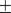 0.1

The numbers are the average values, over the 28 days of the AIRAC cycle, between the partitions and the error bars are standard deviations. Numbers in boldface refer to the partition that maximizes the corresponding metric.

As for the navpoint network, the presence of plateaus for multi-resolution modularity, the values of “randomizing robustness” (bottom part of Table 4), and the small standard errors reveal the robustness of the partitions. Moreover, as before, Infomap provides the most robust communities.

In conclusion, the partitions inferred by the different methods, although relatively close to the existing partitions of the European airspace, are distinct from them. A new design for the European airspace based on these unsupervised detected partitions could be more optimized because the new ACCs or FABs would be more densely connected inside and have less interface (links) with the adjacent ones. It could also help devising dedicated coordination tools and procedures by identifying the boundaries with high traffic exchange volumes.

### The airport network

The last network we consider is the airport network. As explained in the Introduction, this is probably the most studied air traffic network, also for its relation with socio-economic phenomena, such as passenger mobility and epidemic spreading. For this reason we do not present a detailed analysis of the network metrics. In accordance with previous studies [2]–[14] we found that the distributions of degree and strength have a power law tail. This reveals the presence of hubs, i.e. nodes with a high degree and a high strength which shorten significantly the paths on the network (in terms of number of nodes). This feature is also revealed when one studies the relationship between the betweenness centrality and the degree. A summary of the empirical analysis of the airport network obtained from the data investigated here can be found in Ref. [14].

If the network metrics of the airport network have been extensively studied, to the best of our knowledge there are few studies [5], [18] which have considered the community structure of this network. Following the previous analysis on sectors and navpoints, we study here the relationship between this structure and existing division of airspace. We will see that the interpretation of the communities in the airport network is somehow different from the previous one concerning sectors and napvoints.

#### Communities

We consider the network of airports. In this graph, airports are nodes and a link between two of them exists if the two nodes are connected (in the investigated time window) by one or more flights. The link is then weighted according to the number of flights travelling between the two nodes. Here the European network has between 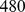 and 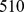 nodes, depending on the day, and around 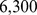 directed edges.

**Figure 7:**
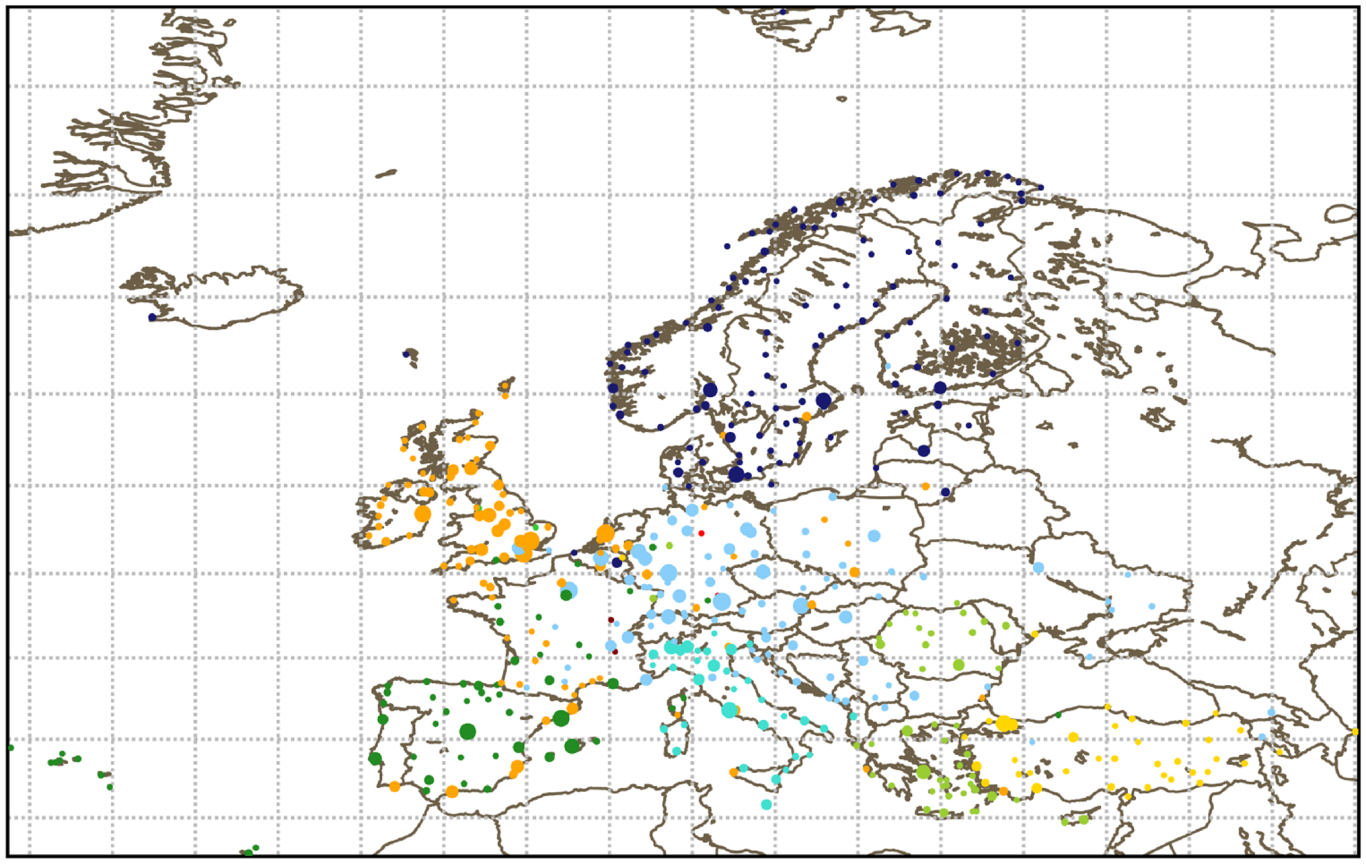
European network of airports on May 6, 2010. Each circle is an airport, its radius proportional to its strength. Each community, detected with the modularity method, is represented by a different color. The links between nodes have been omitted for readability.

An example of a partition obtained with the modularity method is presented in Fig. 7. As one can see, the typical size of a community is supranational, roughly the same as a FAB. The communities are mainly geographical with the majority of nodes close to each other in a single community. Moreover, the borders of the communities seem to be more or less consistent with the national borders. Still, some nodes are far away from their communities.

The number of communities for each algorithm is presented in Table 5. The modularity algorithm gives the biggest partition, even bigger than the FABs. Instead, OSLOM and Infomap give between twice and thrice this number, but still less than the number of NAs. In the second line of the table we show the minimum number of communities (induced or existing) which include 90% of the nodes in the network. As for the sector network, we can see that 50% of communities contains 10% of nodes in the case of the NA partitioning. When the value of 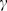 of the multi-resolution modularity is chosen in order to maximize MI with the two existing partitions, one obtains unsupervised partitions with a number of communities quite close to NA (for 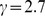) and to FABs (for 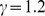). Panel (c) of Fig. 3 shows that the number of airport communities as a function of 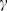 has clear plateaus corresponding to a number of communities equal to 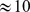 and 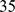, very close to the number of FABs and NA.

**Table 5:** Average number of communities in the network of airports and average minimum number of communities containing 90% of the nodes.

Existing part.	NA	FABs			
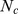	42.0 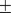 0.	12.0 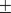 0.			
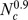	21 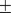 0.4	9.1 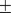 0.3			
Unsupervised	Inf.	Modularity	OSLOM	Mod. max. ( 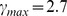 )	Mod. max. ( 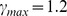 )
part.					
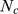	24.5. 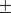 3.9	9.4 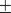 1.2	16.3 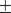 2.7	31.9 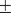 7.0	16.9 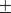 7.2
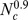	12.0. 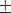 1.8	7.2 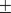 0.4	10.2 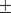 1.0	17.3 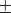 3.1	10.4 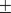 2.9

The top part refers to existing partitions and the bottom part to the unsupervised partitions. Results were obtained averaging over 28 days, with standard deviations taken as error bars.

Table 6 shows a summary of the comparison between unsupervised partitions and existing partitions. In all cases the multi-resolution modularity outperforms the other methods in terms of the similarity with the existing partitions. The modularity gives competitive outcome for FABs because the optimal value of 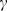 is 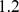, which is very close to 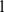 (and both values belong to the same plateau, see Fig. 3c).

**Table 6:** Comparisons of the partitions of the airport network by using the Rand index, the Mutual information, and the robustness.

Rand Index	Infomap	Modularity	OSLOM	Mod. max. ( 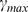 )	
NA	0.17 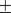 0.07	0.33 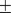 0.03	0.37 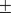 0.04	**0.50** 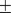 **0.03**	2.7 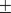 0.7
FAB	0.22 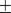 0.05	0.41 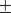 0.03	0.36 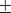 0.04	**0.42** 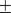 **0.02**	1.2 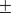 0.3
**Mutual Inf.**	Infomap	Modularity	OSLOM	Mod. max. ( 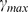 )	
NA	0.41 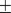 0.05	0.42 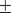 0.02	0.45 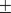 0.04	**0.61** 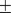 **0.02**	2.7 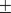 0.7
FAB	0.47 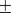 0.04	0.53 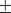 0.02	0.50 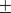 0.03	**0.55** 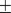 **0.02**	1.2 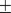 0.3
**Robustness**	Infomap	Modularity	OSLOM	Mod. max. ( 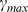 )	
NA	**0.97** 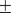 **0.03**	0.88 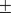 0.04	0.81 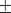 0.04	0.74 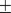 0.04	2.7 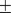 0.7
FAB				0.81 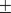 0.08	1.2 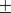 0.3

The numbers are the average values, over the 28 days of the AIRAC cycle, between the partitions and the error bars are standard deviations. Numbers in boldface refer to the partition that maximizes the corresponding metric.

To have a more precise idea of the partitions and see if they adhere to the FABs partition, we study the over-expression of nationalities in the communities of each partition. The results are shown in Tables 7 and 8. The communities detection highlights some of the main features of the future FAB partition. UK and Ireland, as well as Spain and Portugal, are always in the same community, which is a FAB. The northern countries are also together, although they are not exactly aggregated the same way in all partitions. The central Europe is more problematic. In the current design, France and Germany are in the same FAB (see Fig. S5). However from Fig. 6 and the corresponding one for OSLOM communities it is clear that France and Germany are in separated communities. In the over-expression analysis of Tables 7 and 8 France and Germany are in fact never together, with the exception of Infomap, which has a very large community. Finally, while Italy and Greece are together in the same FAB, there is no partition where they are in the same community. In fact, probably due to its geographical location, Italy is in its own community according to two unsupervised partitions (Modularity and OSLOM). On the contrary, it is striking to see that Greece can be in the same community as much as Romania and Turkey are together with Ukraine, whereas Greece and Turkey themselves are never in the same one. This might be due to the diplomatic issues between the two countries. Moreover, the community aggregating Turkey, Azerbaijan and Georgia is visible in all partitions. This community could be a good choice for a FAB if these countries were willing to officially declare one.

**Table 7:** Over-expressions of nationality in different partitions.

Modularity				OSLOM			
Id	Size	Country	Frac.	Id	Size	Country	Frac.
0	114	Finland	0.94	0	78	Germany C.	0.56
		Denmark	1.0			Austria	1.0
		Norway	1.0	1	77	UK	0.88
		Sweden	0.95			Ireland	1.0
1	97	UK	0.90	2	73	Finland	0.94
		Ireland	1.0			Sweden	0.90
2	90	Germany C.	0.69	3	45	Spain	0.73
3	59	Spain	0.85			Portugal	0.77
		Portugal	0.92	4	44	Turkey	0.92
4	49	Greece	0.97	6	41	Norway	0.76
		Romania	1.0			Denmark	0.5
5	38	Turkey	0.92	7	33	Italy	0.72
6	33	Italy	0.86	8	27	Greece	0.79
				9	18	Romania	0.73

Over-expressions of nationality in different partitions: for each partition, we display the ids of the communities, their sizes, the countries over-expressed within it and the fraction of the countries' airports included in the communities. These partitions have been obtained for May, 6 2010.

**Table 8:** Over-expressions of nationality in different partitions (second part).

FABs				Infomap			
Id	Size	Country	Frac.	Id	Size	Country	Frac.
0	90	Germany C.	1.0	0	246	Germany C.	0.97
		France	1.0			Spain	1.0
1	69	Greece	1.0			France	0.90
		Italy	1.0			Italy	1.0
2	66	Norway	1.0	1	38	Turkey	0.94
		Finland	1.0	2	30	Sweden	0.72
3	58	UK	1.0	3	29	Greece	0.93
		Ireland	1.0	4	20	Norway	0.44
4	47	Denmark	1.0	5	17	Romania	1.0
		Sweden	1.0	6	14	Norway	0.31
5	46	Spain	1.0	7	12	Finland	0.75
		Portugal	1.0	8	10	Norway	0.22
6	41	Turkey	1.0	9	9	Ukraine	0.78
7	22	Croatia	1.0	10	9	Denmark	0.88
		Austria	1.0	11	8	UK	0.16
8	18	Romania	1.0	12	8	Portugal	0.62
9	13	Poland	1.0	13	7	Estonia	1.0
	13	Lithuania	1.0	14	5	Serb. & Mont.	0.8
10	10	Ukraine	1.0				
11	6	Serb. & Mont.	1.0				

Second part of Table 7.

The Infomap partition is also very different from the others. Indeed, it shows a massive community, aggregating most countries in western Europe, including Ireland, UK, France, Benelux, Germany, Spain, Portugal, Italy, and Switzerland, as well as countries in central Europe, including Austria, Croatia, Slovakia, Czech Republic, Slovenia, Hungary, and Poland. The community is in fact so big that some of these countries, even if they are totally included in it, are not over-expressed: the probability to find an airport of a given country within the community or in the whole Europe is almost the same. Only a few countries – Greece, northern countries, Romania, Turkey – are not in this cluster, as well as a few western, small airports (less than 10). The massive community is present in several days of the AIRAC cycle, but not everyday.

Note also that, like for the other networks, every measure of robustness, in particular those shown at the bottom of Table 6, indicate that the obtained partitions are indeed robust.

All these results seem to validate the general idea of the FABs, even if their actual boundaries could be different if based on the unsupervised community detection. However, the whole idea of inferring the FABs based on the communities of airports raises some issues. First, in our analysis, airports are different from sectors or navigation points because they do not represent a part of the airspace where the trajectories are managed. Of course, as a first approximation, one can consider that the traffic between airports give the main flow for the airspace. Second point, the airports can have long range interactions, and thus be a priori in the same community, while in two different countries which might not be in the same FAB. Hence, it is important to highlight these long range interactions and see how they are interfering with the rather “compact” communities we found in the network of airports.

#### Extracting the role of distance in the airport network

In transportations systems like the airspace, nodes tend to be more connected to their geographical neighbors, just because they are close to each other. But of course there are other non local causes to the formation of communities. For instance, Ryanair has dedicated airports all over Europe, which are more likely to be connected. Hence, Beauvais in France (close to Paris) or Ciampino in Italy (close to Rome) are more likely to be connected to each other than to Fiumicino or Charles de Gaulle, respectively.

A way of capturing this phenomenon and avoiding the distance bias has been explained in Section “Methodology” and is based on the method described in Ref. [32]. The idea is to maximize modularity by using a null hypothesis that takes into account the geographical distance. From our analysis we find that the deterrence function 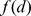 of Eq. 3 is unimodal, being zero for small distances and reaching a maximum around 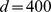 km and decaying slowly to zero. Since the estimation of the deterrence function depends on the number of bins used for 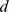, we choose 100 bins since this value gives a good tradeoff between a too coarse description, obtained with few bins, and a too noisy one, obtained when the number of bins is too large.

**Figure 8:**
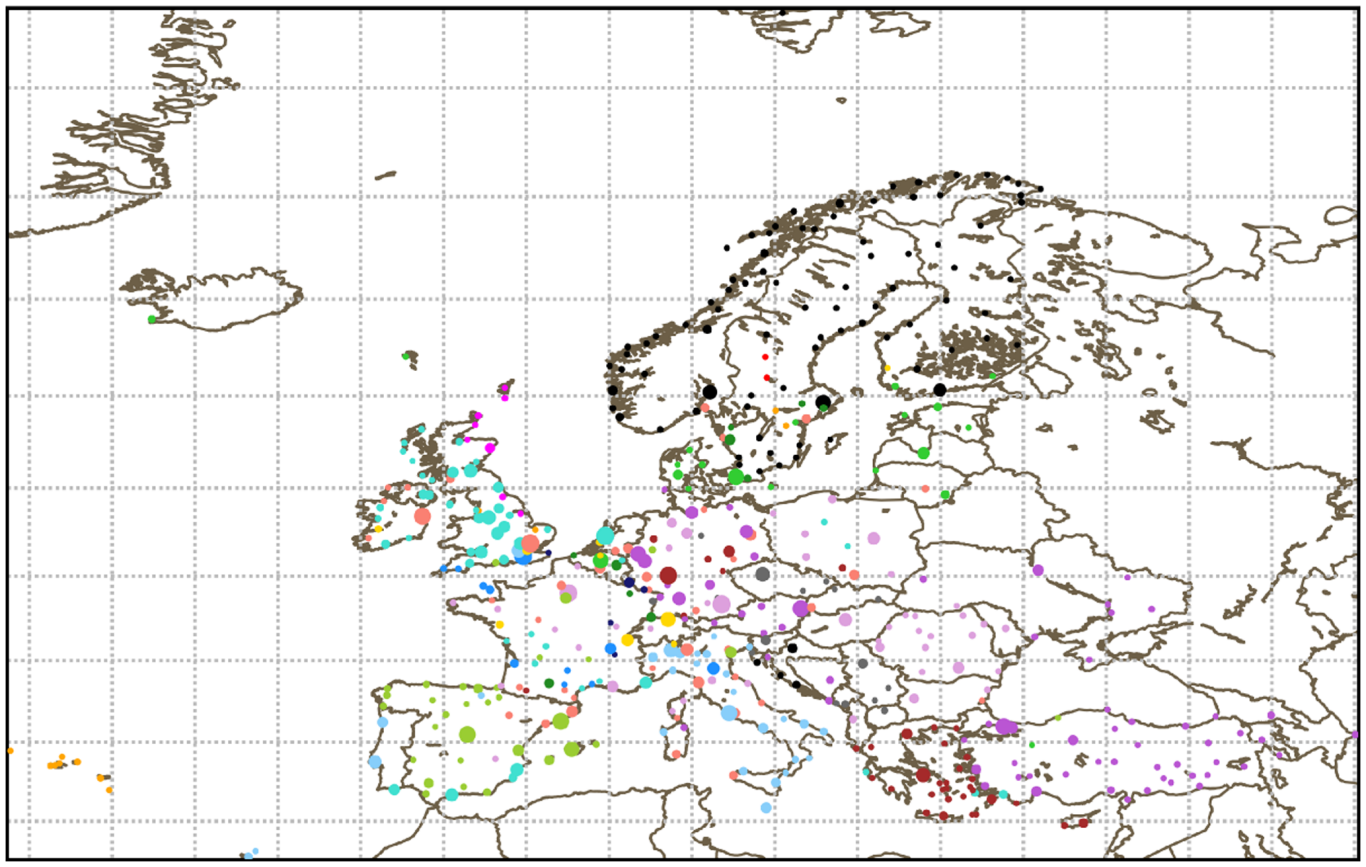
Communities of the airport network obtained by maximizing the modularity with the spatial null model of Eq. (3). The maximization is performed by using a simulated annealing process. Each circle is an airport, its radius proportional to its strength.

The result of this community detection is displayed in Fig. 8. As a point of comparison, one could consider the partition of Fig. 7, which shows the modularity partition (with the usual null model) in the same day. The difference between the two Figures is thus only in the null model (and the maximization algorithm). The picture with the geographical null model is very different. The communities are much less geographically constrained and long range interactions between airports are enhanced. For instance, the European airports operated mainly by Ryanair are now in the same community (displayed in salmon on the map). These interactions between these airports cannot be explained by geographical distance between them but only by the fact that they are operated by the same company. This community is not in the previous Fig. 7 and has been detected only by removing the distance bias.

In the same line, we can notice the specialization of airports surrounding big cities. Paris Orly for instance is in the same community than many Spanish airports, which is consistent with its actual role. Paris Charles De Gaulle is more linked to the central and eastern Europe and Beauvais, as noticed before, is operated only by Ryanair and is in its network. Around London each airport is also in a different community. Luton is more linked to other English airports, whereas Heathrow is linked to Italy and Portugal, Gatwick and the London City Airport to airports located in France and Switzerland mainly, and Stansted is in the Ryanair network.

Geographical clusters did not disappear completely. Spain, United Kingdom, Italy, Greece and Scandinavian countries have their own community, despite the spatial null model. This might be due to national characteristics (language, common firms, etc.) which are not directly concerned by distance. These additional characteristics can be embodied in the formation of dedicated regional airlines which in turn create these local structures. Moreover, the presence of bottlenecks, observed for instance in the northern countries, can explain the clusters. Indeed, starting from a small airport, one has to go through Stockholm, Oslo or Helsinki to exit the area. Hence, regardless of the distance, all these airports are in the same community.

In conclusion the unsupervised detections of communities show two distinct characteristics of the network of airports. First, it has a strong geographical basis, with airports more likely to be in the same community if they are close to each other. Moreover, the typical size of communities and also the geographical boundaries are consistent with the future partition of the European airspace. Secondly, the airports show some long distance interactions, which are not explained by geographical proximity, and cannot be strictly linked to the operational partitions.

Another potential application to these detections may be to build direct dedicated communication tools between airports that show a strong “long-range interaction” to smooth and optimize the coordination – as already being addressed by some existing SESAR projects.

## Conclusions

We have investigated the structure of the ECAC airspace at different scales: the navigation point scale, the sector scale and the airport scale. By representing the system at each scale with a network, we have performed a community detection analysis in order to detect the groups of elements in the system that have homogeneous behaviour with respect to the actual air traffic conditions. Specifically, we have presented results relative to different unsupervised community detection algorithms that provide meaningful partitions of the airspace, starting from the mere knowledge of the actual air traffic flows.

At each scale the community detection algorithms provide useful insights on the system. Generally speaking, as shown in Fig. 3, the multi-resolution algorithm is capable of reproducing the three levels associated to the existing partitions by using different values of the resolution parameter 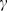. Even if the method identifies the same number of hierarchical levels as in real data, the identified communities are often quite different from the existing ones. Since the unsupervised communities are obtained from the traffic data, they are a hierarchical partition better reflecting the use of the airspace. In general, all the obtained partitions seem to be quite robust. When we compute the partitions over the different AIRAC days we get a number of communities that is not very variable over the days. i.e. standard deviations are quite low. Moreover, the multi-resolution algorithm is able to find a number of communities which is constant over a certain range of 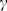.

More specifically:

- In the case of the navpoint network, we have obtained that the modularity partition gives big communities whose size is comparable to the NAs, while the OSLOM one gives a finer clusterization, close to the ACCs. It is worth mentioning that the multi-resolution algorithm with 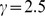 performs quite well for ACCs and with 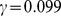 for NA, both in terms of number of communities and of MI. In the latter case the partition is the one obtained with classic modularity, since 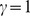 and 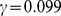 belong to the same plateau. The partitioning obtained by using the Infomap algorithm is close to reproduce the air traffic sectors.
- In the case of sector network, the Infomap and OSLOM partitions are quite close to the NAs, at least in terms on number of communities. The multi-resolution algorithm with 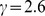 well reproduces NAs in terms of MI. The multi-resolution algorithm with 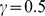 well reproduces FABs in terms of number of communities and it is the best among the investigated methods in terms of MI. However, it is worth mentioning that already the modularity partition seems to give the same results as the modularity partition with 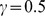. The relatively small value of MI in these two cases would indicate that the boundaries of the obtained partitions are distinct from the real FABs ones. This might suggest a possible alternative design of the FABs, which preserves their total number. The Infomap also results to be the most robust method.
- For the airport network, the multi-resolution algorithm with 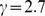 and 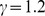 well reproduces FABs and NAs, respectively, in terms of number of communities, of MI, and of RI. The Infomap results to be again the most robust method. When the community detection algorithm takes into account the geographical constraints, as in Section “Extracting the role of distance in the airport network”, then some long distance interactions between airports emerge. These are not explained by geographical proximity, and cannot be strictly linked to general operational aspects. Rather, they might reveal strategies operated by specific airlines, such as, for example, Ryanair. In fact, Ryanair has not a business model based on large hubs. Rather, flight plans are scheduled without explicitly considering the presence of connection flights.

All these results underline the difference between the bottom-up approach with an unsupervised partition based on the traffic and the top-down approach of the real construction of the existing partitions. The different algorithms used are therefore able to capture different features of the airspace organization and, in some cases, they might provide alternative ways of redesigning already present pieces of the airspace. The new ACCs, for instance, would be indeed more densely connected inside and have less interface (links) with the exterior, which is an added value from an operational point of view. Another potential application is the use of these methods to highlight the boundaries (between sectors, ACCs, NAs, FABs) that require intensive coordination, as they may deserve dedicated coordination tools and procedures. Also, the establishment of direct communication links between closely-connected distant airports, as identified by the community detection, could be interesting.

The obtained partitions provide alternative ways of designing the future European airspace. Indeed, it is important to emphasize that Europe (as well as the US) is moving toward a new scenario of air traffic management according to an ambitious and long term project termed SESAR [21] aiming at changing the architecture of the European airspace based on a new set of paradigms [41]. In this new scenario cross national control units will be defined and they will be based more significantly on traffic demand than on national constraints. In this framework our bottom up approach for the partitioning of the European airspace with community detection algorithms could be used to improve the design of this important transport infrastructure.

We believe also that this approach could be fruitfully adopted in other types of traffic networks [42].

## Supporting Information

Figure S1 **Communities of the navpoint network based on the national airspaces.**
(TIF)

Figure S2 **Communities of the navpoint network based on the control centres.**
(TIF)

Figure S3 **Communities of the sector network based on the functional airblocks.**
(TIF)

Figure S4 **Communities of the sector network based on the national airspaces.**
(TIF)

Figure S5 **Communities of the sector network based on the control centres.**
(TIF)

Figure S6 **Communities of the airport network based on the functional airblocks.** Each circle is an airport, its radius proportional to its strength.
(TIF)

Figure S7 **Communities of the airport network based on the national airspaces.** Each circle is an airport, its radius proportional to its strength.
(TIF)

Information SI **Existing partitions present in the different networks, due to FABs, NAs, and ACCs subdivision.**
(PDF)
