# Supplementary figures and images for: Multi-Scale Analysis of the European Airspace Using Network Community Detection
Source: PLoS One. 2014 May 8;9(5):e94414. doi: 10.1371/journal.pone.0094414 (PMC4014470; doi:10.1371/journal.pone.0094414)

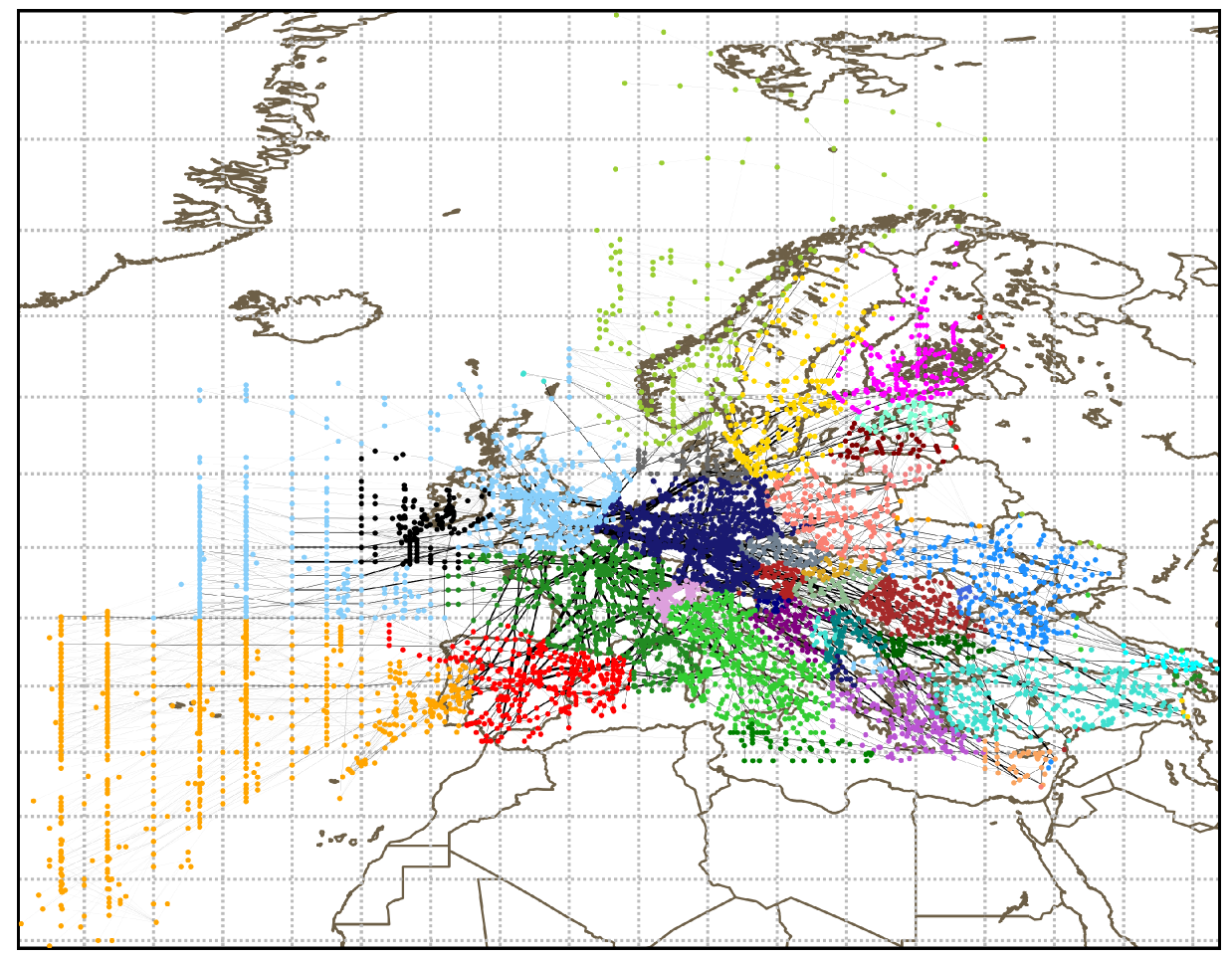

Supplement: Figure S1 — Communities of the navpoint network based on the national airspaces. (TIF) [file pone.0094414.s001.tif]

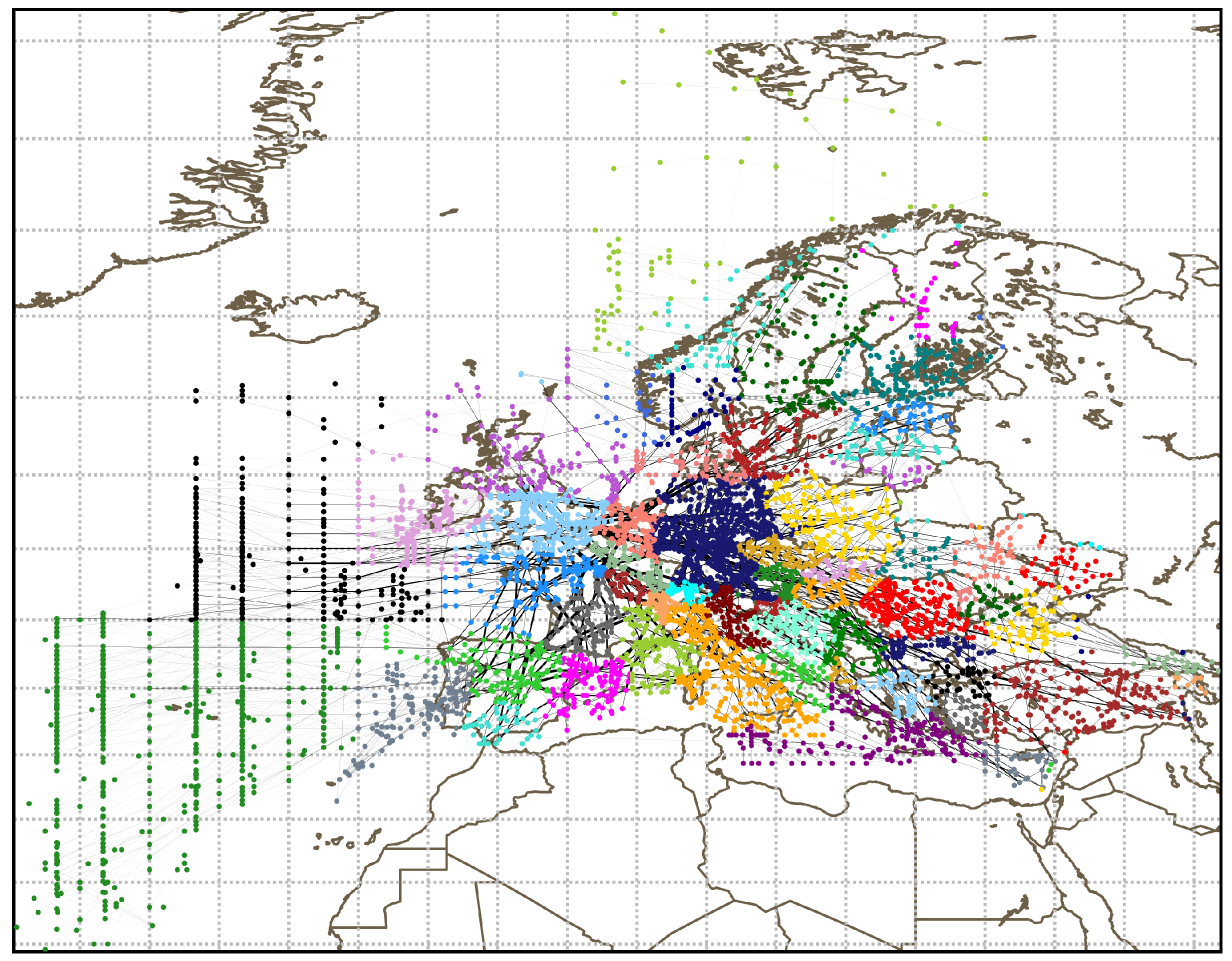

Supplement: Figure S2 — Communities of the navpoint network based on the control centres. (TIF) [file pone.0094414.s002.tif]

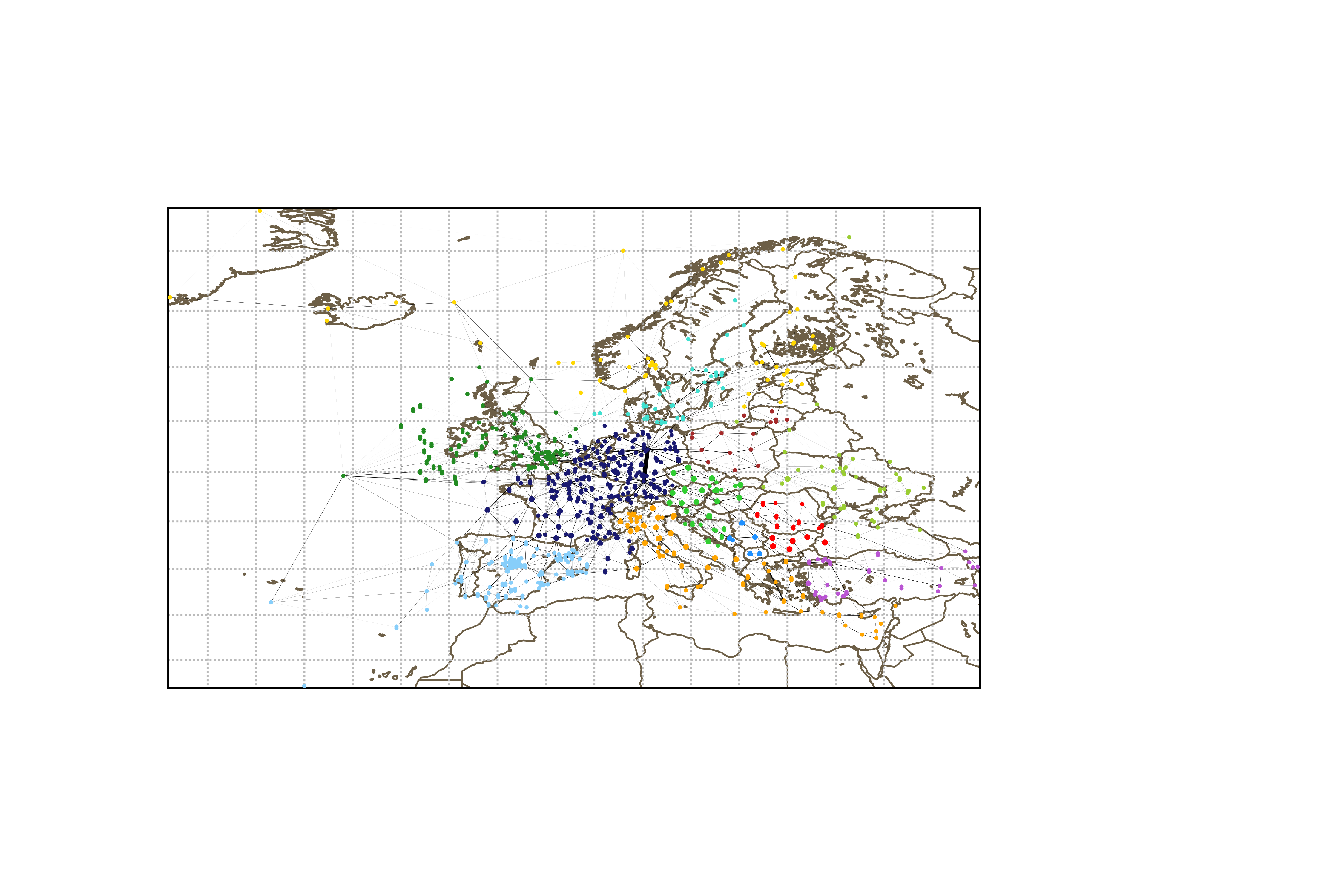

Supplement: Figure S3 — Communities of the sector network based on the functional airblocks. (TIF) [file pone.0094414.s003.tif]

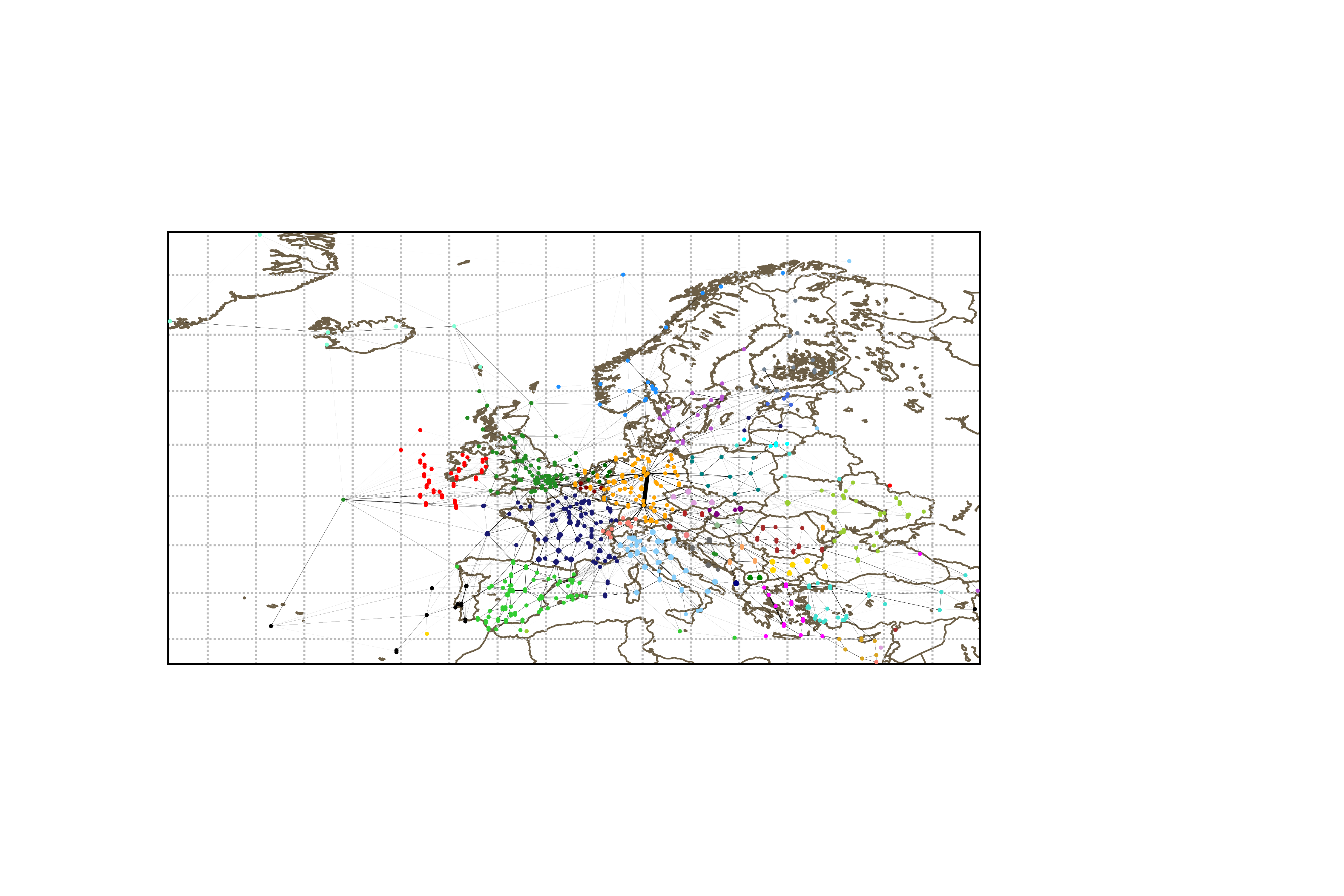

Supplement: Figure S4 — Communities of the sector network based on the national airspaces. (TIF) [file pone.0094414.s004.tif]

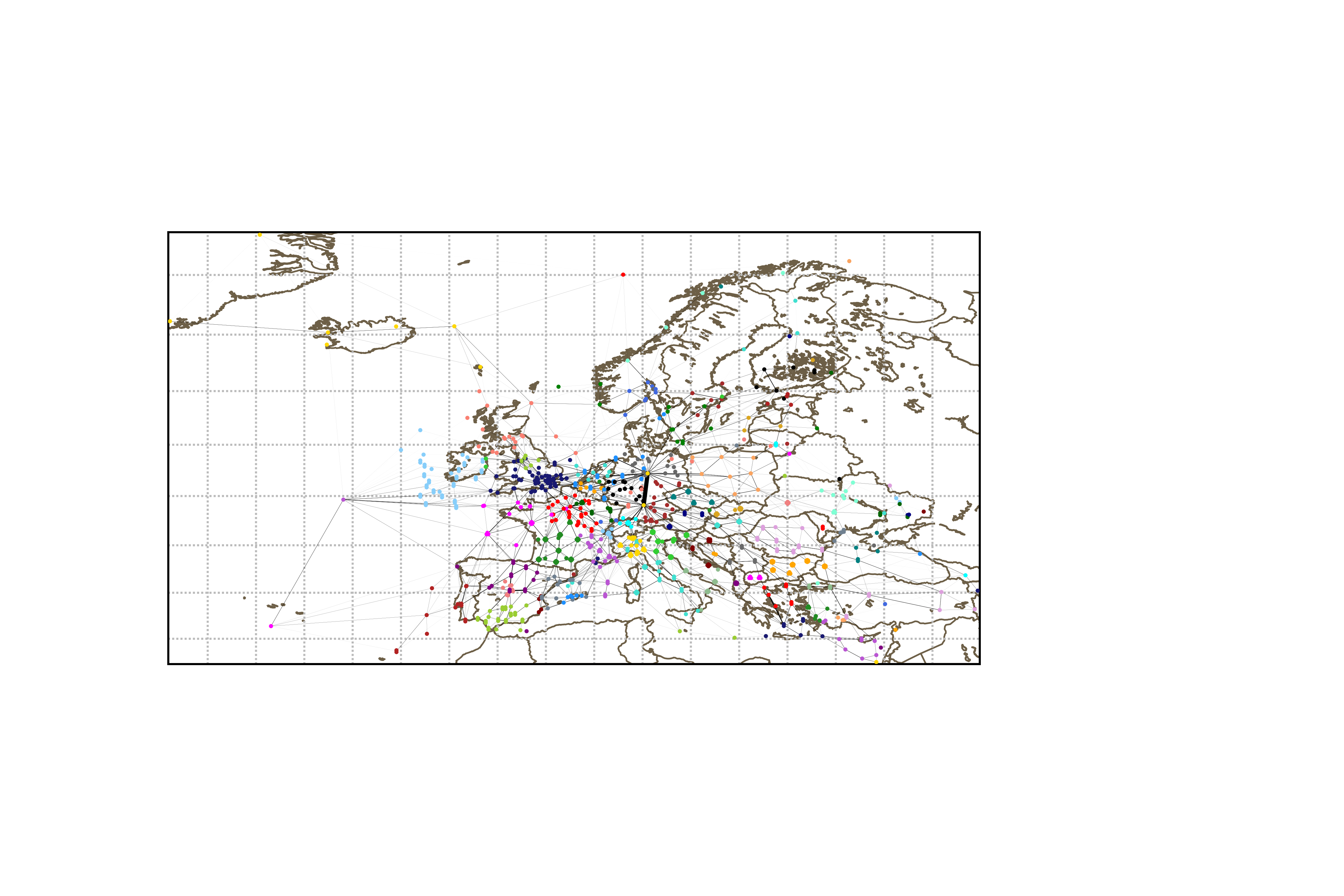

Supplement: Figure S5 — Communities of the sector network based on the control centres. (TIF) [file pone.0094414.s005.tif]

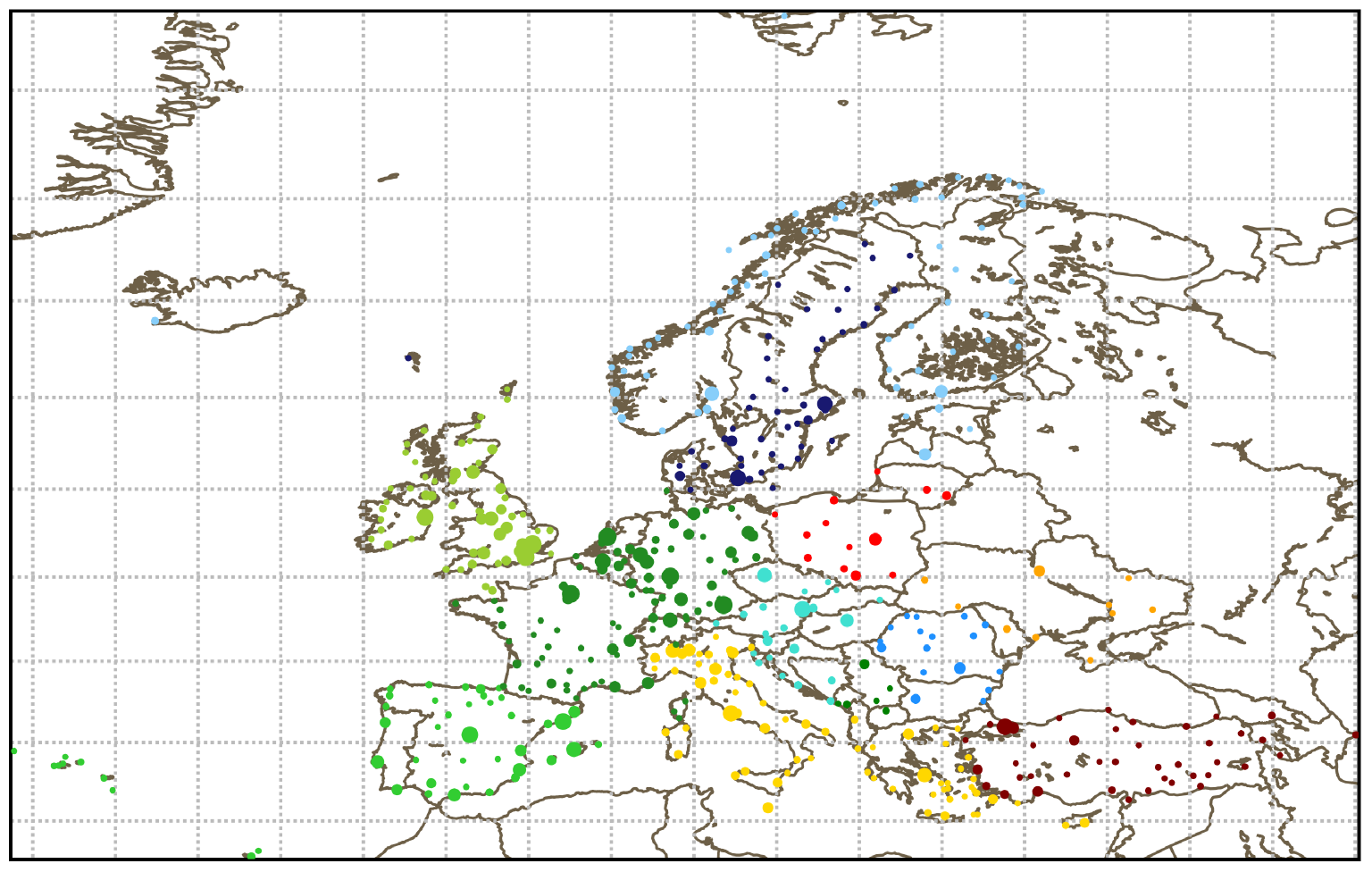

Supplement: Figure S6 — Communities of the airport network based on the functional airblocks. Each circle is an airport, its radius proportional to its strength. (TIF) [file pone.0094414.s006.tif]

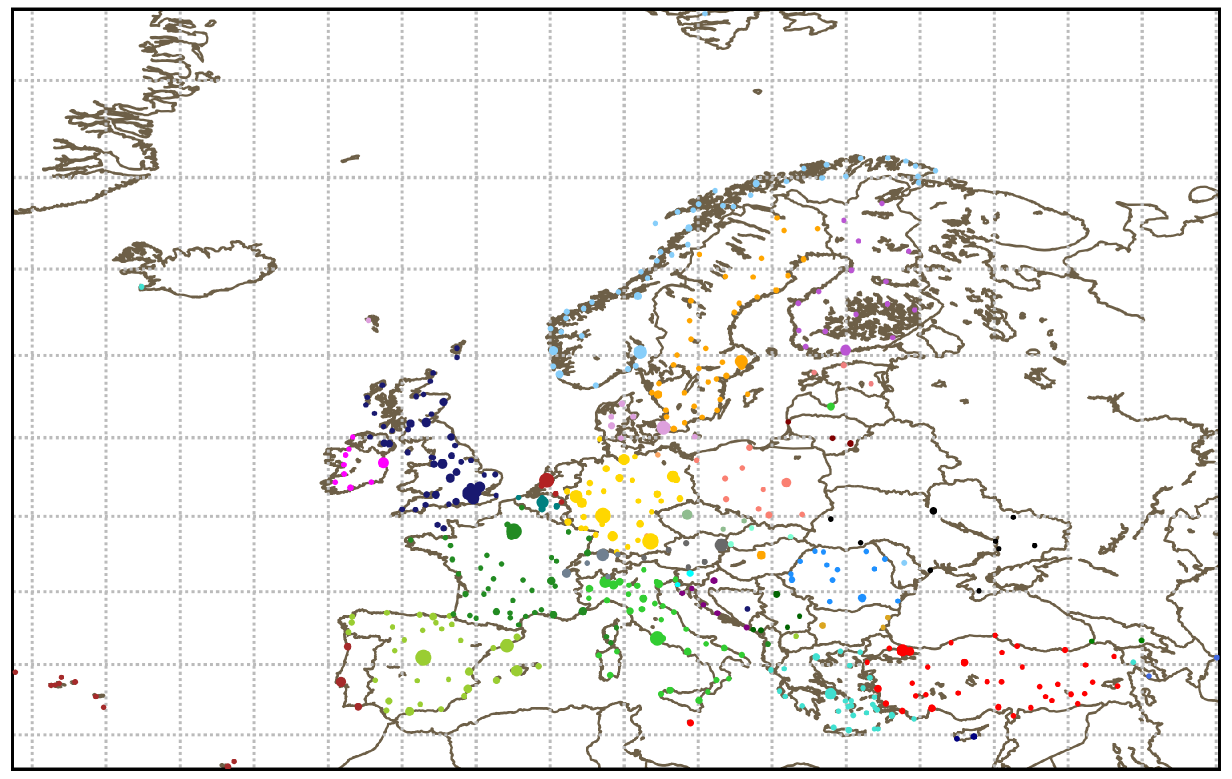

Supplement: Figure S7 — Communities of the airport network based on the national airspaces. Each circle is an airport, its radius proportional to its strength. (TIF) [file pone.0094414.s007.tif]
