# Supplementary material for: Multi-Scale Analysis of the European Airspace Using Network Community Detection
Source: PLoS One. 2014 May 8;9(5):e94414. doi: 10.1371/journal.pone.0094414 (PMC4014470; doi:10.1371/journal.pone.0094414)
Supplement: Information SI — Existing partitions present in the different networks, due to FABs, NAs, and ACCs subdivision. (PDF) [file pone.0094414.s008.pdf]

Existing partitions

The FABs, or Functional AirBlocks, are described in Table S1. We split the description in two parts. The first set contains the FABs which are already officially defined. The other set contains groups of countries (East Europe, Turkey, Serbia) defined by the authors according to geographical and cultural proximity in order to have a complete tessellation of ECAC airspace. Thus these are not actual FABs.

Here is a series of figures showing the different existing partitions of the systems.

- Figure S1 shows the communities of the navpoint network based on the national airspaces;
- Figure S2 shows the communities of the navpoint network based on the control centers;
- Figure S3 shows the communities of the sector network based on the functional airblocks (FABs);
- Figure S4 shows the communities of the sector network based on the national airspaces;
- Figure S5 shows the communities of the sector network based on the control centers;
- Figure S6 shows the communities of the airport network based on the functional airblocks;
- Figure S7 shows the communities of the airport network based on the the national airspaces.

Figure Legends

Figure 1. Figure S1: Communities of the navpoint network based on the national airspaces.

Figure 2. Figure S2: Communities of the navpoint network based on the control centres.

Figure 3. Figure S3: Communities of the sector network based on the functional airblocks.

Figure 4. Figure S4: Communities of the sector network based on the national airspaces.

Figure 5. Figure S5: Communities of the sector network based on the control centres.

Figure 6. Figure S6: Communities of the airport network based on the functional airblocks. Each circle is an airport, its radius proportional to its strength.

Figure 7. Figure S7: Communities of the airport network based on the national airspaces. Each circle is an airport, its radius proportional to its strength.

Tables

Table 1. Composition of FABs

Official FABs	Countries included
SW FAB	Spain, Portugal
FAB EC	France, Switzerland, Monaco, Belgium, Germany, Netherlands, Luxembourg
Blue MED	Italy, Cyprus, Greece, (Egypt, Tunisia), Albania, (Jordan)
FAB CE	Czech Republic, Slovakia, Austria, Hungary, Croatia, Slovenia, Bosnia and Herzegovina
DANUBE	Romania, Bulgaria
NEFAB	Estonia, Finland, Latvia, Norway
UK-IR FAB	United Kingdom, Ireland
Danish-Swedish FAB	Denmark, Sweden
BALTIC FAB	Poland, Lithuania

Other FABs	Countries included
EAST Europe FAB	Ukraine, Moldova, part of Russia
Turkey FAB	Armenia, Turkey, Azerbaijan, Georgia
Serbia FAB	Serbia and Montenegro, Macedonia

Composition of the official Functional Airblocks (FABs) and the ones we defined ourselves.
